# Resveratrol reprograms ARG1⁺ microglia to remodel the immunosuppressive microenvironment in primary CNS lymphoma via secretion cytokine

**DOI:** 10.1186/s12974-026-04004-w

**Published:** 2026-08-18

**Authors:** Chenghan Chen, Ming Lu, Zibo Du, Ziqi Shen, Yao Liu, Jingwen Fu, Yiwen Wang, Wangming Zhang, Ping Zhang, Qi Luo, Jun Wang

**Affiliations:** 1 Department of Neurosurgery, Nanfang Hospital Southern Medical University, Guangzhou, 510515 China; 2 Department of Neurosurgery, The National Key Clinical Specialty, Guangdong Provincial Key Laboratory on Brain Function Repair and Regeneration, The Neurosurgery Institute of Guangdong Province, Zhujiang Hospital Southern Medical University, Guangzhou, 510280 China; 3 Guangdong Sanjiu Brain Hospital, Guangzhou, 510515 China; 4 Guangdong Provincial Key Laboratory of Chinese Medicine Pharmaceutics, School of Traditional Chinese Medicine, Southern Medical University, Guangzhou, 510515 China

**Keywords:** PCNSL, Tumor microenvironment, Microglia, ARG1

## Abstract

Primary central nervous system lymphoma (PCNSL) is a CNS-restricted lymphoma with poor prognosis. Despite sharing pathological features with systemic diffuse large B-cell lymphoma (DLBCL), PCNSL exhibits unique molecular mutation patterns, highlighting the critical role of the intracranial tumor microenvironment (TME). However, the role of microglia within TME remains poorly understood. Here, integrative analysis of single-cell transcriptomic data from systemic DLBCL and PCNSL reveals a distinct TME in PCNSL characterized by a heightened immunosuppressive state via microglia. Here, we identify a subpopulation of ARG1⁺ microglia enriched in the PCNSL tumor core, representing activated morphology and reduced branching complexity. Functionally, these microglia exhibit enhanced cytokine secretion via upgrading PI3K-AKT signal pathway, thereby remodeling the TME and ultimately promoting tumor progression. Targeting this microglia subtype with resveratrol, a bioactive small molecule, resulted in significant tumor suppression in vivo and vitro, accompanied by reduced expression of immunosuppressive cytokines and functional reprogramming of microglia. Collectively, our findings identify ARG1⁺ microglia as key orchestrators of the immunosuppressive TME in PCNSL through secretion cytokine and highlight microglia-targeted modulation by resveratrol as a potential therapeutic strategy.

**Supplementary Information:**

The online version contains supplementary material available at https://doi.org/10.1186/s12974-026-04004-w.

## Introduction

Primary central nervous system lymphoma (PCNSL) is a rare and aggressive subtype of diffuse large B-cell lymphoma (DLBCL) that is confined to the central nervous system (CNS), and it is biologically and clinically distinct from its systemic counterpart due to its unique anatomical and immunological environment [1–5]. Over the past decades, numerous clinical trials have evaluated both conventional and novel therapeutic agents for PCNSL. Nevertheless, high-dose methotrexate (HD-MTX) remains the cornerstone of frontline therapy [6–8]. Despite initial responsiveness, more than 60% of patients experience disease relapse or develop resistance to methotrexate, leading to poor overall survival [2, 9, 10]. Although over 95% of PCNSL cases are histopathologically classified as DLBCL, significant differences in treatment response, recurrence patterns, and survival outcomes between PCNSL and systemic DLBCL support the unique biological behavior of PCNSL within the CNS [11–15]. These discrepancies strongly suggest that the CNS microenvironment plays a pivotal role in shaping the pathophysiology and treatment resistance of PCNSL [4, 15].

Microglia, the resident immune cells of the CNS, maintain immune surveillance and rapidly respond to microenvironmental changes through phagocytosis and cytokine secretion [16–20]. While transient activation can be neuroprotective, persistent stimulation may induce chronic functional reprogramming [21]. In tumors, microglia often acquire immunosuppressive phenotypes that promote tumor progression and immune evasion; however, the classical M1/M2 classification is insufficient to fully describe microglial states within the tumor microenvironment [22–24].

In this study, we established intracranial PCNSL models using patient-derived primary cells and a PCNSL-specific cell line [25], recapitulating the pathological features and poor prognosis of clinical PCNSL. Comparative analyses with systemic DLBCL models revealed enrichment of ARG1⁺ disease-associated microglia (DAM), suggesting that the CNS-specific immune microenvironment promotes immunosuppressive remodeling in PCNSL. Furthermore, we identified resveratrol as a potential therapeutic compound that suppresses ARG1⁺ microglia and inhibits tumor progression with minimal systemic toxicity.

## Methods

### Single-cell RNA sequencing data processing and integrative analysis

Publicly available single-cell RNA sequencing (scRNA-seq) datasets of primary central nervous system lymphoma (PCNSL) and systemic diffuse large B-cell lymphoma (DLBCL) were collected and jointly analyzed. Single-cell RNA sequencing analysis. Publicly available single-cell RNA sequencing datasets of PCNSL and DLBCL were obtained from the Gene Expression Omnibus (GEO), including GSE182434 (DLBCL) and GSE203552 (PCNSL). The DLBCL dataset comprised seven patient samples with over 13,000 single cells, whereas the PCNSL dataset included two patient samples with over 10,000 single cells. Data preprocessing and downstream analyses were performed using the Seurat package. Cells expressing fewer than 500 genes or with a mitochondrial gene percentage > 10% were excluded. No additional doublet removal algorithm was applied; instead, standard Seurat quality-control procedures and subsequent clustering were used to remove low-quality or ambiguous cells. Gene expression data were normalized using the LogNormalize method with a scale factor of 10,000, followed by identification of the 3,000 most variable genes using the FindVariableFeatures function (selection.method = “vst”) and data scaling using the default ScaleData settings. Principal component analysis (PCA) was performed using 40 principal components. Batch effects were corrected using the Harmony algorithm with the grouping variable Tumor_type and default parameters. The first 30 Harmony-corrected dimensions were used for UMAP visualization and downstream clustering analyses. Raw count matrices were processed using the Seurat (v4.0 or later) framework in R. Cells with low quality were filtered out based on standard criteria, including low gene counts, excessive mitochondrial gene expression, or potential doublets. Gene expression values were normalized and log-transformed prior to downstream analyses. To integrate PCNSL and DLBCL datasets while minimizing batch effects arising from technical and biological heterogeneity, we applied the Harmony algorithm following principal component analysis (PCA). Harmony-corrected embeddings were used for subsequent clustering and visualization analyses.

### Immune signature–based non-negative matrix factorization

To dissect immune-related heterogeneity across the integrated dataset, we curated immunologic gene signatures from the Molecular Signatures Database (MSigDB) C7 collection. Expression matrices restricted to these immune-related gene sets were subjected to Non-negative Matrix Factorization (NMF) using established algorithms. Based on cophenetic correlation coefficients and stability metrics, the optimal factorization rank was determined, stratifying cells into four immune meta-clustered subtypes, designated M1–M4. Uniform Manifold Approximation and Projection (UMAP) was performed using Harmony-corrected dimensions to visualize the distribution of M1–M4 subtypes across the integrated single-cell landscape. Cell-type composition analysis was conducted to compare the relative abundance of each meta-cluster between PCNSL and DLBCL samples. To determine the optimal factorization rank for non-negative matrix factorization (NMF), candidate ranks (k = 2–10) were evaluated based on reconstruction error, residual sum of squares (RSS), and module correlation structure. Each rank was repeated five times to assess clustering stability. Reconstruction error decreased markedly up to k = 4 and plateaued thereafter, while the marginal reduction in RSS dropped below 5% beyond k = 4, indicating diminishing improvement with increasing model complexity. Hierarchical clustering of module correlations further demonstrated that four meta-modules exhibited high intra-module correlation and low inter-module correlation. Collectively, these analyses supported k = 4 as the optimal rank for NMF decomposition.

### Pathway enrichment and immune functional characterization

To investigate immune pathways underlying the differences between meta-clustered subtypes, Gene Set Variation Analysis (GSVA) was performed using a curated panel of nine immune-related pathways, encompassing T-cell activation, immune checkpoint signaling, macrophage polarization, and microglial activation. GSVA enrichment scores were calculated at the single-cell level and aggregated across subtypes. Heatmaps and box plots were used to visualize pathway activity differences among M1–M4 clusters. Correlation and clustering analyses were further applied to assess the relationship between microglial activation and immunosuppressive signatures, including pathways associated with M2 macrophage infiltration and immune inhibitory molecule expression.

### Marker gene expression analysis

To further characterize the biological features of each immune meta-cluster, expression levels of key functional markers were examined. Violin plots were generated to compare the distribution of proliferation-related genes (e.g., MKI67), tumor suppressor genes (e.g., TP53), and immune interaction markers critical for B-cell and T-cell communication (e.g., MS4A1 [CD20] and CD40) across M1–M4 subtypes. These analyses were used to infer differences in malignant potential and immune suppression status between PCNSL- and DLBCL-enriched clusters.

### Cell lines and culture

HMC3, U2932, and HKBML are used in this study. HMC3 (Procell, Wuhan, China) is cultured in Eagle’s Minimum Essential Medium (MEM; Gibco) containing with 10% fetal bovine serum (FBS; Gibco) and 1% penicillin-streptomycin (P/S; Gibco). U2932 (Procell, Wuhan, China) is cultured in RPMI-1640 medium (Gibco) with 10% FBS and 1% P/S. HKBML (YajiCell, Shanghai, China) is cultured in Dulbecco’s Modified Eagle Medium/Nutrient Mixture F-12 (DMEM/F12; Gibco) supplemented with 20% FBS and 1% P/S. All cell lines are incubated at 37 °C in a humidified atmosphere with 5% CO₂ and are routinely tested to confirm the absence of mycoplasma contamination (Table 1).

### Primary PCNSL cell culture

Primary PCNSL cells were obtained from intraoperative and biopsy-derived tumor specimens collected from 24 patients diagnosed with PCNSL in Zhujiang hospital, Guangzhou Sanjiu hospital and Nanfang hospital. Tumor specimens were collected from 24 patients with PCNSL, including both stereotactic biopsy and surgical resection samples. Primary tumor cell cultures were successfully established from five patients. Among these, primary cells from two independent patients successfully generated intracranial patient-derived xenograft (PDX) models following stereotactic implantation into female BALB/c nude mice, and these two independent PDX models were used for subsequent patient-derived experiments. Immediately after surgical excision or biopsy, tumor tissues were transported on ice in PBS. Under sterile conditions, tissue specimens were minced into small fragments (~ 1 mm³) using sterile surgical scissors and mechanically dissociated without enzymatic digestion. The tissue fragments were individually transferred into wells of 96-well plates, with each well containing a single or a few fragments to promote clonal expansion. Wells were carefully monitored over time for the outgrowth of tumor cells. Medium containing DMEM/F12 and Neurobasal supplemented with B27, glutamate and 1% penicillin-streptomycin, which was refreshed every 2–3 days. Primary cells were cultured in 37 °C with 5% CO₂ until sufficient cell proliferation was observed. Due to the limited availability and clinical value of patient-derived samples, primary PCNSL cells were used exclusively for in vivo experiments, such as intracranial xenograft modeling, to evaluate tumorigenicity and therapeutic response.

### Intracranial PCNSL model in vivo

To establish an orthotopic PCNSL model, a stereotactic intracranial injection technique is employed. Immunodeficient BALB/c nude mice (4–6 weeks old) are anesthetized with 2% isoflurane and securely positioned in a stereotaxic apparatus (RWD Life Science). After scalp disinfection and a midline incision, a small burr hole is drilled at the following coordinates: 1.8 mm lateral to the right of bregma, 1.0 mm anterior, and 3.5 mm in depth from the dura mater. A total of 1 × 10⁶ PCNSL cells suspended in 5 µL of PBS are slowly injected into the right caudate-putamen over a period of 5 min using a 10 µL Hamilton syringe. To minimize reflux, the needle is left in place for an additional 5 min before being gradually withdrawn. The incision is sutured, and mice are placed on a heating pad for recovery. Mice are monitored daily for survival, neurological symptoms, and general health status. Body weight is measured every 2–3 days post-surgery using an electronic balance. Rapid weight loss, along with neurological deficits and behavioral abnormalities, is used as a humane endpoint. Resveratrol was administered in vivo via oral gavage (i.g.). Briefly, resveratrol was prepared as a suspension in normal saline at a concentration of 1 mg/mL. Mice received 0.2 mL of the suspension per administration. Female BALB/c nude mice were randomly assigned to the experimental groups before stereotactic intracranial implantation. Six mice were included in each experimental group. MRI image analysis, histological evaluation, and data quantification were performed by investigators blinded to the group allocation. Humane endpoints were defined as body weight loss exceeding 20% of the initial body weight or the development of severe neurological symptoms preventing normal feeding or drinking. Mice that died within 2 days after stereotactic implantation due to causes unrelated to tumor progression were excluded from the analysis. No other animals met the exclusion criteria.

### Immunofluorescence staining

Mice are euthanized using an overdose of isoflurane, followed by intracardiac perfusion with cold phosphate-buffered saline (PBS, Gibco) and 4% paraformaldehyde (PFA, Biosharp). Brains are harvested and post-fixed in 4% PFA for 24 h at 4 °C, then cryoprotected in 15% sucrose solution until they sink, followed by immersion in 30% sucrose solution. Tissues are embedded in optimal cutting temperature (OCT) compound (Tissue-Tek, Sakura Finetek) and sectioned at 30 μm thickness using a cryostat (Leica CM1950).

Sections are washed with PBST (PBS with 0.1% Triton X-100) for 15 min and blocked with 5% normal donkey serum (Servicebio) for 30 min at room temperature. Primary antibodies are applied and incubated overnight at 4 °C in PBS. The primary antibodies used include anti-Iba1 (Abcam, 1:1000), anti-ARG1 (Proteintech, 1:500), and anti-CD20 (Abcam, 1:1000), anti-inos (Abcam, 1:1000), anti-CD206 (Proteintech, 1:1000) (Table 2). After incubation for more than 16 h, sections are washed and incubated with Alexa Fluor-594–conjugated secondary antibodies (Invitrogen, 1:500) and Alexa Fluor-488–conjugated secondary antibodies (Abclonal, 1:1000) for 30 min at room temperature. Nuclei are counterstained with DAPI (Thermo Fisher). Finally, sections are mounted using anti-fade mounting medium and imaged with a confocal microscope (Zeiss LSM 980). Three-dimensional reconstruction and colocalization analyses are performed using Imaris software (Bitplane).

### Hematoxylin and Eosin (H&E) staining

For histopathological evaluation, major organs (heart, liver, spleen, lung, and kidney) are harvested and fixed in 4% paraformaldehyde (PFA) for 48 h. Following fixation, tissues are embedded in paraffin and subjected to a controlled freezing step at − 20 °C to stabilize the blocks. The temperature is then gradually equilibrated to room temperature. Paraffin blocks are sectioned at a thickness of 5 μm and mounted onto glass slides. For antigen retrieval, tissue sections are treated with sodium citrate buffer and allowed to cool naturally to room temperature. Subsequently, slides are deparaffinized in xylene, rehydrated through a graded ethanol series, and stained with hematoxylin for 5 min followed by eosin for 2 min. After dehydration and clearing, slides are examined under a bright-field microscope for pathological assessment.

### 7.0T MRI scanning of mouse brain

To monitor intracranial tumor growth in vivo, magnetic resonance imaging (MRI) was performed using a 7.0T small-animal MRI system (Bruker BioSpec 70/30, Germany). Mice were anesthetized with 2% isoflurane in oxygen and positioned on the MRI bed with a dedicated mouse brain coil. T2-weighted images were acquired using a fast spin-echo sequence (TR/TE = 2500/30 ms, field of view = 20 × 20 mm, slice thickness = 0.5 mm). Imaging was conducted at multiple time points (day 7, 14, and 21 post-tumor implantation). Tumor volumes were calculated using ImageJ by summing the tumor area across sequential slices and multiplying by slice thickness.

### Novel object recognition test

The novel object recognition (NOR) test is conducted to assess recognition memory in mice. Animals first undergo habituation in an open-field arena for two consecutive days. On the third day, two identical objects are introduced into the arena for familiarization. After a 24-hour retention period, one of the familiar objects is replaced with a novel item. The duration of exploration directed toward each object is recorded. Discrimination index (DI) and recognition index (RI) are calculated to evaluate memory performance.

### Open field test

The open field test is applied to evaluate spontaneous locomotor activity and anxiety-related behavior. Each mouse is placed individually into the center of a square testing arena and allowed to explore freely for 5 min. Locomotion patterns are recorded using a video tracking system. The total distance traveled, as well as the time and distance spent in the central versus peripheral zones, are measured for behavioral analysis.

### Quantitative Real-Time PCR (qRT-PCR)

Total RNA is isolated from cultured cells using the Total RNA Extraction Kit (Magen, China) according to the manufacturer’s protocol. The concentration and purity of RNA are assessed using a NanoDrop 2000 spectrophotometer (Thermo Fisher Scientific, USA). Complementary DNA (cDNA) is synthesized from 1 µg of total RNA using the PrimeScript RT Reagent Kit (Takara, Japan). Quantitative real-time PCR (qPCR) is carried out with TB Green^®^ Premix Ex Taq™ II (Takara) on a QuantStudio 6 Flex Real-Time PCR System (Applied Biosystems, USA). The amplification conditions include an initial denaturation at 95 °C for 30 s, followed by 40 cycles of denaturation at 95 °C for 5 s and annealing/extension at 60 °C for 30 s.

### Cell co-culture system

To investigate microglia–lymphoma cell interactions, a Transwell co-culture system with 0.4 μm pore inserts (Corning) is used. HMC3 microglial cells are seeded in the lower chamber of 6-well plates at a density of 5 × 10⁵ cells per well and allowed to adhere for approximately 16 h. The following day, either HKBML or U2932 lymphoma cells (5 × 10⁵ cells/well) are added to the upper chamber in complete medium. Co-cultures are maintained for 24 h at 37 °C in a humidified incubator with 5% CO₂. After incubation, microglia from the lower chamber are collected for subsequent analyses, including quantitative real-time PCR and immunofluorescence. In selected experiments, resveratrol (20 µM) is supplemented at the initiation of co-culture to evaluate its regulatory effects on microglial activation and ARG1 expression.

### Single-cell dissociation of mouse brain tissue

To obtain microglia from mouse brains, single-cell suspensions are prepared using a neural tissue dissociation kit (Neural Tissue Dissociation Kit (P), RWD Life Science, China) following the manufacturer’s protocol. Briefly, freshly harvested brains are minced in cold HBSS and enzymatically digested at 37 °C with continuous gentle agitation for 30 min. The digested tissue is then mechanically triturated and passed through a 70 μm cell strainer to remove undigested fragments. The resulting cell suspension is washed twice with PBS and resuspended in MACS buffer (PBS supplemented with 0.5% BSA and 2 mM EDTA) for subsequent magnetic labeling and microglia isolation.

### Magnetic separation of CD11b⁺ microglia

CD11b⁺ microglia are enriched from freshly prepared single-cell suspensions using magnetic-activated cell sorting (MACS) with anti-CD11b MicroBeads (Miltenyi Biotec, Germany), in accordance with the manufacturer’s instructions. The cell suspension is incubated with MicroBeads targeting CD11b for 15 min at 4 °C. Following incubation and washing, the labeled cells are applied to MS columns placed within a magnetic separator. CD11b⁺ cells are retained in the magnetic field, while unlabeled cells are washed through. After removing the column from the magnet, CD11b⁺ cells are flushed out and collected. Purified microglia are immediately processed for downstream assays, including qRT-PCR, immunofluorescence staining, or RNA sequencing.

### RNA sequencing and bioinformatic analysis

Bulk RNA sequencing is performed on microglial RNA samples using the Illumina NovaSeq 6000 platform, generating 150 bp paired-end reads. Raw sequencing data are subjected to quality control using fastp, and clean reads are aligned to the mouse reference genome (GRCm38) using HISAT2. Transcript abundance is quantified using StringTie, and expression levels are normalized as fragments per kilobase of exon per million mapped fragments (FPKM). Differential gene expression analysis is conducted using the DESeq2 package in R. Genes with an adjusted p value < 0.05 and |log₂ fold change| > 1 are considered significantly differentially expressed. Visualization of differential gene expression is performed using ggplot2, including volcano plots and heatmaps. Unsupervised hierarchical clustering is conducted using the pheatmap package based on the top 1,000 variable genes to assess global transcriptomic differences among groups. Principal component analysis (PCA) is performed to visualize transcriptome-level variance. Functional enrichment analyses, including Gene Ontology (GO) and Kyoto Encyclopedia of Genes and Genomes (KEGG) pathway analysis, are performed using the clusterProfiler R package. Protein–protein interaction (PPI) networks of selected gene sets are constructed using the STRING database and visualized with Cytoscape.

### Western blot analysis

After euthanasia, mouse brains were collected and homogenized using RIPA lysis buffer (Beyotime, China) supplemented with a protease inhibitor cocktail (Roche, Switzerland) on ice for 30 min. Lysates were centrifuged at 12,000 × g for 15 min at 4 °C, and the supernatant was collected for protein quantification using a BCA Protein Assay Kit (Beyotime, China). Samples were incubated at 37 °C for 30 min, and absorbance was measured at 562 nm using a microplate reader (BioTek, USA). Protein concentrations were calculated based on the standard curve.Equal amounts of protein (20 µg per sample) were separated by 12.5% SDS-PAGE and transferred onto PVDF membranes (Millipore, USA). Membranes were blocked with 5% nonfat milk in TBST for 1 h at room temperature and then incubated overnight at 4 °C with primary antibodies, including anti-ARG1 (Proteintech, 1:1000) and anti-β-actin (Affinity, 1:5000) as an internal control. After washing three times with TBST (5 min each), membranes were incubated with HRP-conjugated secondary antibodies (goat anti-rabbit or goat anti-mouse IgG, 1:5000, Affinity) for 1 h at room temperature. Protein bands were visualized using an enhanced chemiluminescence (ECL) substrate (Beyotime) and imaged with a ChemiDoc imaging system (Bio-Rad, USA).Band intensity was quantified using ImageJ software (NIH, USA), and the relative expression of target proteins was normalized to β-actin. Full length western blots are included in the supplementary.

### Enzyme-Linked Immunosorbent Assay (ELISA)

To determine the secretion levels of inflammatory cytokines in the tumor microenvironment, commercial ELISA kits (Elabscience, Wuhan, China) were used according to the manufacturer’s instructions. The detected cytokines included tumor necrosis factor-α (TNF-α), interleukin-6 (IL-6), interleukin-1β (IL-1β), and interleukin-2 (IL-2) (Table 1). Briefly, cell culture supernatants or mouse brain tissue homogenates were collected and centrifuged at 3000 × g for 10 min to remove debris. Standards and samples were added to the antibody-precoated wells and incubated at 37 °C for 90 min. After washing, biotinylated detection antibodies and HRP-conjugated streptavidin working solution were sequentially added. Following several washing steps, TMB substrate solution was added for color development for 10–15 min, and the reaction was terminated with stop solution. Absorbance was measured at 450 nm using a microplate reader (BioTek, USA).Cytokine concentrations were calculated according to the standard curve and expressed as pg/mL. All samples were assayed in triplicate to ensure data reliability.

**Table 1:** Catalogue numbers for reagents

Reagents	Source	Identifier
Resveratrol	MCE	Cat# HY-16,561
4,6-diamidino-2-phenylindole (DAPI)	Abcam	Cat# ab104139
Minimum Essential Medium (MEM)	Gibco	Cat# 11,095,080
Fetal Bovine Serum (FBS)	Gibco	Cat# 10,099,141
penicillin-streptomycin (P/S)	Gibco	Cat# 15,140,122
RPMI 1640	Gibco	Cat# 11,875,093
Dulbecco’s Modified Eagle Medium/Nutrient Mixture F-12 (DMEM/F12)	Gibco	Cat# 11,320,033
B27	Gibco	Cat# 17,504,044
Phosphate-buffered saline (PBS)	Gibco	Cat# 10,010,023
4% Paraformaldehyde (PFA)	Biosharp	Cat# BL539A
Optimal cutting temperature (OCT)	Tissue-Tek	Cat# 4583
Normal donkey serum	Servicebio	Cat# G1217
Mouse IL-1β(Interleukin 1 Beta) ELISA Kit	Elabscience	Cat# E-EL-M0037
Mouse IL-6(Interleukin 6) ELISA Kit	Elabscience	Cat# E-EL-M0044
Mouse TNF-α(Tumor Necrosis Factor Alpha) ELISA Kit	Elabscience	Cat# E-EL-M3063
Mouse IFN-γ(Interferon Gamma) ELISA Kit	Elabscience	Cat# E-EL-M0048
Prestained Protein Ladder	Affinity	Cat# KF8007

### Cell viability assay (CCK-8)

Cells (5 × 10⁵ per well) are seeded into 96-well plates and allowed to adhere for 24 h. resveratrol or vehicle control is then added and incubated for 48 h. Cell viability is assessed using the Cell Counting Kit-8 (CCK-8, Dojindo) according to the manufacturer’s instructions. Absorbance at 450 nm is measured using a microplate reader, and results are expressed as mean ± SEM from triplicate wells.

### Multiplex immunofluorescence staining

Multiplex immunofluorescence (mIHC) staining was performed to characterize the activation states and phenotypic polarization of microglia in different tumor models (Table 2). Mouse brains were harvested at the indicated time points, fixed in 4% paraformaldehyde overnight at 4 °C, cryoprotected in 30% sucrose, and embedded in optimal cutting temperature (OCT) compound. Coronal brain Sects.  (10–14 μm) were prepared using a cryostat and mounted onto adhesive glass slides.Sections were air-dried, rehydrated in phosphate-buffered saline (PBS), and subjected to antigen retrieval using citrate buffer (pH 6.0) at 95–98 °C for 15–20 min. After cooling to room temperature, endogenous peroxidase activity was quenched by incubation with 3% hydrogen peroxide for 10 min. Non-specific binding was blocked with 5% normal serum supplemented with 0.3% Triton X-100 for 1 h at room temperature. Primary antibodies were applied sequentially and incubated overnight at 4 °C. After thorough washing, signal amplification was performed using a tyramide signal amplification (TSA)-based mIHC system according to the manufacturer’s instructions. Briefly, horseradish peroxidase (HRP)-conjugated secondary antibodies were applied, followed by incubation with fluorophore-conjugated tyramide substrates. Between each staining cycle, antibody stripping was achieved by heat-mediated antigen retrieval to prevent cross-reactivity while preserving deposited fluorophores. After completion of all staining cycles, nuclei were counterstained with DAPI, and sections were mounted using antifade mounting medium. Negative controls were included by omitting primary antibodies.

**Table 2:** Catalogue numbers for antibodies

Antibodies	Source	Identifier
Rabbit anti-Iba1	Abcam	Cat# ab178846
Mouse anti-Iba1	Abcam	Cat# ab283319
Rabbit anti-CD206	Proteintech	Cat# 83485-1-RR
Rabbit anti-inos	Abcam	Cat# ab178945
Rabbit anti-CD20	Proteintech	Cat# 24828-1-AP
Mouse anti-CD20	Proteintech	Cat# 60271-1-Ig
Rabbit anti-ARG1	Proteintech	Cat# 16001-1-AP
Rabbit anti-β-actin	Affinity	Cat# AF7018
Goat Anti-Rabbit IgG (H + L) HRP	Affinity	Cat# S0001
Alexa Fluor-594 anti-mouse secondary antibodies	Invitrogen	Cat# A-21,209
Alexa Fluor-488 anti-rabbit secondary antibodies	Abclonal	Cat# AS035

### Isolation of resveratrol

The fermentation of strain GLQ04 was performed on a rice solid medium statically (each hexagonal bottle autoclaved and contained 50 g of rice, 0.25 g yeast extract, 0.25 g dextrose and 75 mL of water) in 250 × 700 mL hexagonal bottle for 30 days at room temperature. The fermented rice culture was soaked with 80% methanol and concentrated under reduced pressure, then extracted 3 times with EtOAc to give an extract of 114.8 g. The resulting residue was portioned into 10 fractions (Fr.1 − Fr.10) by using MCI gel CHP 20P with a gradient of aqueous MeOH (20%–100%).

Fr.2 was separated by using RP-18 eluted with aqueous MeOH (20%–50%) to yield four fractions (Fr.2.1–Fr.2.4). Fr.2.1 was isolated by Sephadex LH-20 (MeOH) to get six fractions (Fr.2.1.1–Fr.2.1.6). Fr.2.1.2 was subjected to semi-preparative HPLC (MeOH/H2O, 40:60, flow rate: 3 mL/min) followed by semi-preparative HPLC (ACN/H2O, 24:76, flow rate: 3 mL/min) to obtain 1 (6.3 mg, tR = 19 min, flow rate: 3 mL/min). Fr.2.1.3 was subjected to semi-preparative HPLC (MeOH/H2O, 32:68, flow rate: 3 mL/min) followed by semi-preparative HPLC (ACN/H2O, 33:67, flow rate: 3 mL/min) to obtain 2 (3.4 mg, tR = 14 min, flow rate: 3 mL/min); followed by semi-preparative HPLC (ACN/H2O, 24:76, flow rate: 3 mL/min) to obtain 6 (4.7 mg, tR = 18 min, flow rate: 3 mL/min) and 7 (3.9 mg, tR = 18 min, flow rate: 3 mL/min); followed by semi-preparative HPLC (ACN/H2O, 24:76, flow rate: 3 mL/min) to obtain 7 (4.7 mg, tR = 18 min, flow rate: 3 mL/min) and 8 (6.0 mg, tR = 17 min, flow rate: 3 mL/min).

Fr.3 was separated by using RP-18 eluted with aqueous MeOH (20%–100%) to yield four fractions (Fr.3.1–Fr.3.4). Fr.3.2 was isolated by Sephadex LH-20 (MeOH) to get three fractions (Fr.3.2.1–Fr.3.2.3). Fr.3.2.1 was subjected to preparative HPLC (MeOH/H2O with 1‰ FA, 40:60, flow rate: 9 mL/min) followed by semi-preparative HPLC (ACN/H2O with 1‰ FA, 34:66, flow rate: 3 mL/min) to obtain 10 (6.3 mg, tR = 19 min, flow rate: 3 mL/min); followed by semi-preparative HPLC (ACN/H2O, 32:68, flow rate: 3 mL/min) to obtain 11 (6.3 mg, tR = 20 min, flow rate: 3 mL/min).

Fr.5 was separated by Sephadex LH-20 (MeOH) to generated four fractions (Fr.5.1–Fr.5.4). Fr.5.2 was subjected to semi-preparative HPLC (MeOH/H2O, 55:45, flow rate: 3 mL/min) followed by semi-preparative HPLC (ACN/H2O, 46:54, flow rate: 3 mL/min) to obtain 19 (2.5 mg, tR = 24.0 min, flow rate: 3 mL/min); followed by semi-preparative HPLC (ACN/H2O, 55:45, flow rate: 3 mL/min) to obtain 15 (2.7 mg, tR = 17.0 min, flow rate: 3 mL/min), 16 (3.9 mg, tR = 17.0 min, flow rate: 3 mL/min) and 18 (3.7 mg, tR = 22.0 min, flow rate: 3 mL/min).

Fr.6 was isolated by Sephadex LH–20 (MeOH) to obtain four fractions (Fr.6.1–Fr.6.4). Preparative HPLC (MeOH/H2O, 68:32, flow rate: 9 mL/min) was carried out on Fr.6.2, a portion of which precipitated to give 4 (26 mg). Then, the other part was subjected to semi-preparative HPLC (ACN/ H2O with 1‰ FA, 58:42, flow rate: 3 mL/min) to obtain 22 (1.6 mg, tR = 20 min, flow rate: 3 mL/min) and 25 (1.2 mg, tR = 19.0 min, flow rate: 3 mL/min). Semi-preparative HPLC (ACN/H2O, 50:50, flow rate: 3 mL/min) was performed to obtain 24 (3.8 mg, tR = 15 min, flow rate: 3 mL/min). Semi-preparative HPLC (ACN/H2O with 1‰ FA, 55:45, flow rate: 3 mL/min) was performed to obtain 21 (22.7 mg, tR = 14 min, flow rate: 3 mL/min). Semi-preparative HPLC (ACN/H2O, 44:56, flow rate: 3 mL/min) was performed to obtain 31 (0.9 mg, tR = 20 min, flow rate: 3 mL/min). Fr.6.1 was combined into 4 components Fr.6.1.1 − Fr.6.1.4 by preparative TLC. Fr.6.1.2 was segmented using a preparative type (MeOH/ H2O, 60:40, flow rate: 9 mL/min). Then, semi-preparative HPLC (ACN/H2O, 50:50, flow rate: 3 mL/min) was performed to obtain 27 (6.1 mg, tR = 14 min, flow rate: 3 mL/min). Semi-preparative HPLC (ACN/H2O, 50:50, flow rate: 3 mL/min) was performed to obtain 29 (1.6 mg, tR = 15 min, flow rate: 3 mL/min). Fr.6.1.3 was segmented using the preparative type (MeOH/ H2O, 60:40, flow rate: 9 mL/min) and obtained 26 (83 mg, tR = 17 min, flow rate: 3 mL/min). Then, semi-preparative HPLC (ACN/H2O with 1‰ FA, 50:50, flow rate: 3 mL/min) was performed to obtain 28 (0.9 mg, tR = 17 min, flow rate: 3 mL/min). Semi-preparative HPLC (ACN/H2O with 1‰ FA, 45:55, flow rate: 3 mL/min) was performed to obtain 30 (1.8 mg, tR = 16 min, flow rate: 3 mL/min).

Fr.7 was separated by using RP-18 eluted with aqueous MeOH (40%–100%) to yield four fractions (Fr.7.1–Fr.7.4). Fr.7.2 was isolated by Sephadex LH-20 (MeOH) to get six fractions (Fr.7.2.1–Fr.7.2.6). Fr.7.2.3 was subjected to preparative HPLC (MeOH/H2O, 40:60, flow rate: 9 mL/min) followed by semi-preparative HPLC (ACN/H2O, 55:45, flow rate: 3 mL/min) to obtain 12 (2.4 mg, tR = 13.0 min, flow rate: 3 mL/min); followed by semi-preparative HPLC (ACN/H2O, 72:28, flow rate: 3 mL/min) to obtain 13 (6.0 mg, tR = 15.0 min, flow rate: 3 mL/min); followed by semi-preparative HPLC (ACN/H2O, 50:50, flow rate: 3 mL/min) to obtain 14 (5.9 mg, tR = 16.0 min, flow rate: 3 mL/min). Fr.G.2.2 was combined into 5 components Fr.G.2.2.1–Fr.G.2.25 by preparative TLC. Fr.7.2.2.2 was subjected to semi-preparative HPLC (MeOH/H2O, 54:46 with 1‰ FA, flow rate: 3 mL/min) to obtain 23 (5.0 mg, tR = 17.0 min, flow rate: 3 mL/min).

Fr.8 was portioned into 10 fractions (Fr.8.1 − Fr.8.5) by using MCI gel CHP 20P with a gradient of aqueous MeOH (80%–100%). Fr.8.2 was isolated by Sephadex LH-20 (MeOH) to get six fractions (Fr.8.2.1–Fr.8.2.4). Fr.8.2.2 was subjected to preparative HPLC (MeOH/H2O with 1‰ FA, 98:2, flow rate: 9 mL/min) followed by semi-preparative HPLC (ACN/H2O, 85:15, flow rate: 3 mL/min) to obtain 33 (6.6 mg, tR = 24.0 min, flow rate: 3 mL/min).

### Statistical analysis

The data are shown as the mean ± SEM of at least three independent experiments. Statistical analyses were performed using GraphPad Prism (version 10.0) unless otherwise specified. Student’s t test was used for comparisons between two groups, and one-way analysis of variance was used for comparisons among more than two groups. The log-rank test was used to determine the statistical significance of Kaplan–Meier survival curves. P value < 0.05 was considered statistically significant. Statistical significance is indicated as *P < 0.05, **P < 0.01, ***P < 0.001, and ****P < 0.0001.

## Results

### Establishment of intracranial PCNSL models reveals poorer prognosis in in vivo

**Fig. 1:**
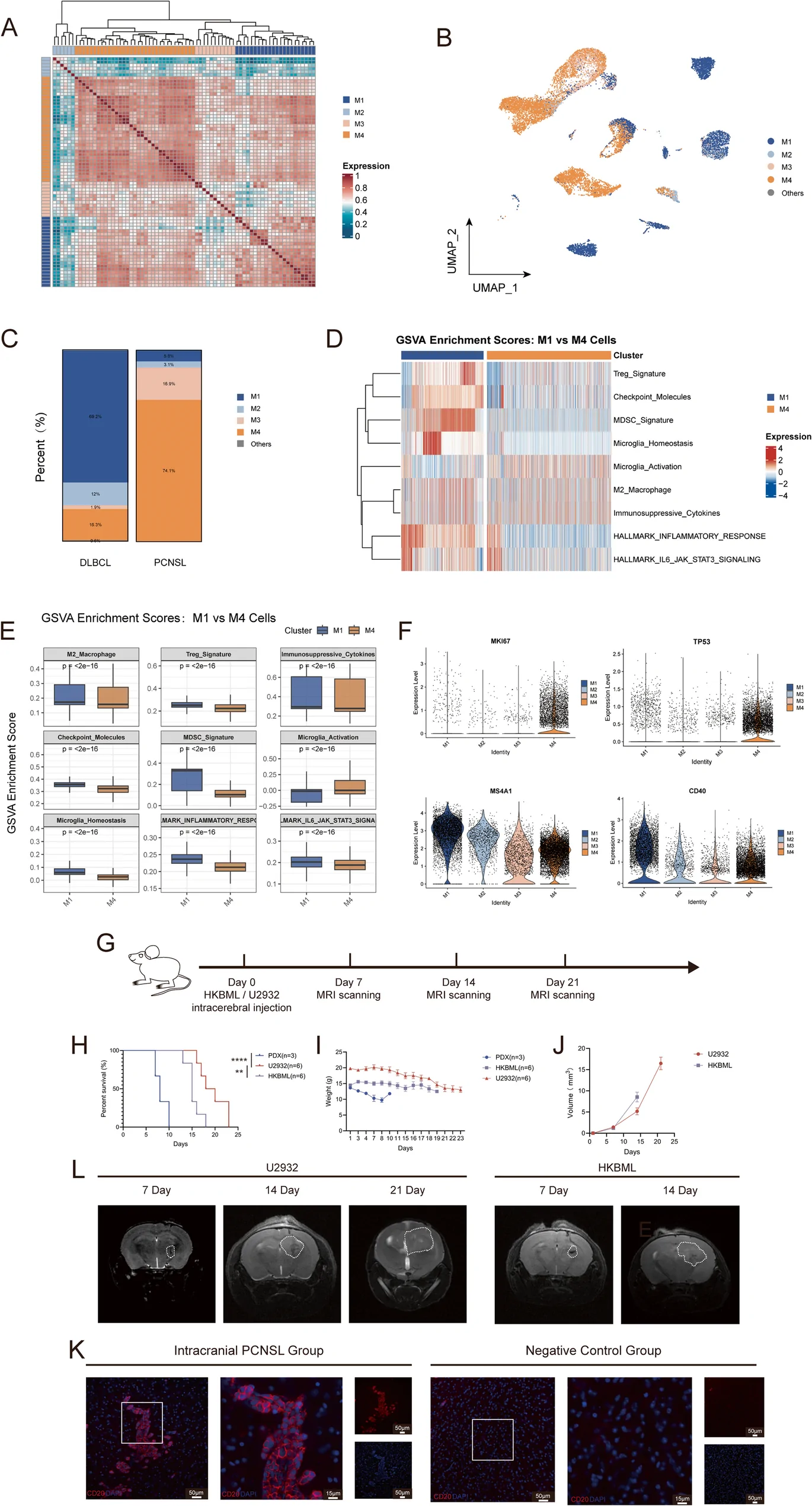
Establishment of Intracranial PCNSL Models Reveals Poorer Prognosis in In Vivo. **a** Identification of four distinct subtypes (M1-M4) through Non-negative Matrix Factorization (NMF) of integrated PCNSL and DLBCL datasets. **b** UMAP visualization depicting the distribution of M1-M4 subtypes. **c** Compositional analysis of M1-M4 subtypes across PCNSL and DLBCL samples. **d** Enrichment of immune-related pathways in M1 versus M4 subtypes revealed by Gene Set Variation Analysis (GSVA). **e** Statistical significance of immune pathway enrichment in M1 versus M4 subtypes, as assessed by GSVA (***, *p* < 0.0001). **f** Expression patterns of the tumor malignancy markers (MKI67, TP53) and key molecules in B-cell immunity (MS4A1, CD40) across the M1-M4 subtypes. **g** Schematic diagram of the PCNSL mice model. **h** Kaplan–Meier survival curves of PCNSL primary cell, HKBML group and U2932 group in vivo (Data represented as mean ± SEM, *n* = 6). **i** Body weight changes in three group mentioned above (Data represented as mean ± SEM, *n* = 6). **j** Quantification of MRI scanning tumor volume of HKBML group and U2932 group(Data represented as mean ± SEM, *n* = 3). **k** Immunofluorescence staining of brain sections from mice injected with PCNSL patient-derived primary cell. Scale bar, 50 μm. Insets show microglia at higher magnification as indicated by the area in white box. Scale bar, 15 μm. **l** Representative images of 7.0T MRI images of mice brains were acquired at days 7, 14, and 21 post-implantation with HKBML group or U2932 group, area within the white dotted line is tumor region. Data are presented as mean ± SEM (*n* = 5 sections per group; 3 biological replicates). Statistical significance was determined using one-way ANOVA followed by Tukey’s multiple comparisons test. *P < 0.05, **P < 0.01, ***P < 0.001, ****P < 0.0001

We first integrate single-cell RNA sequencing data of PCNSL and DLBCL using Harmony to correct for batch effects. Focusing on immunologic signatures from the MSigDB C7 collection, we perform Non-negative Matrix Factorization (NMF), which stratifies the integrated dataset into four distinct meta clustered subtypes M1-M4 (Fig. 1a). The UMAP visualization confirms clear distribution patterns of these M1-M4 cells (Fig. 1b). A compositional analysis reveals that M4 is the most prevalent subtype in PCNSL, whereas M1 is predominant in DLBCL (Fig. 1c), indicating profound biological differences between the two diseases.

To investigate the underlying immune pathways driving this disparity, we conduct Gene Set Variation Analysis (GSVA) on a curated set of nine immune-related pathways, encompassing T-cell activity, immune checkpoint, macrophage polarization, and microglial activation, among others. Both heatmap and box plot visualizations (Fig. 1d and e) demonstrate that among the nine pathways, only microglial activation is significantly enriched in the M4 subtype compared to M1, all others are more active in M1. Furthermore, clustering analysis indicates a strong association between microglial activation and pathways related to immunosuppressive molecules and M2 macrophage infiltration, suggesting a pivotal and potentially immunosuppressive role for microglia in PCNSL, which may be a key factor in CNS-specific immune dysregulation.

Subsequent analysis of key marker expression (Fig. 1f) using violin plots reveal that the proliferation marker MKI67 and the tumor suppressor TP53 are most highly expressed in the M4 subtype, indicating a potentially higher malignant potential in PCNSL. Conversely, expressions of MS4A1 (CD20) and CD40, which are critical for B-cell and T-cell immune interactions, is highest in M1 and lowest in M4. This pattern suggests a state of greater T-cell immune suppression in PCNSL compared to DLBCL.

In previous studies, most intracranial PCNSL models have utilized activated B-cell-like (ABC) DLBCL cell lines such as U2932 or Raji. However, these lines are derived from systemic DLBCL patients and may not fully recapitulate the clinical and biological characteristics of PCNSL. Although over 90% of PCNSL cases are histopathological classified as ABC-DLBCL, the use of non-CNS-derived cell lines limits the translational relevance of existing models. To address this, we employed HKBML cell line—originally established from a PCNSL patient—for the development of an orthotopic intracranial PCNSL model. Nude mice were randomly assigned to receive intracranial injections of an equal number of tumor cells, including HKBML cells, U2932 cells, or patient-derived primary PCNSL cells (Fig. 1g).

Both survival time (Fig. 1h) and body weight (Fig. 1i) demonstrated that mice in the HKBML group showed significantly shorter overall survival and more rapid weight loss compared with the U2932 group. Notably, mice implanted with primary patient-derived PCNSL cells exhibited the poorest survival. MRI analysis further revealed that although tumor volumes were initially comparable between groups, tumors in the HKBML group progressed much more rapidly during later stages (Fig. 1j and l). Due to the aggressive disease progression and shortened survival, longitudinal monitoring of the HKBML group could not be continued beyond the late stage of tumor growth. To validate tumor identity, we performed immunofluorescence staining for the B-cell marker CD20 on brain sections. Tumor cells exhibited enlarged nuclei and strong membrane-localized CD20 expression, confirming successful intracranial engraftment of B-cell lymphoma cells (Fig. 1k). In the intracranial in situ PCNSL model, immunofluorescence analysis reveals that the tumor tissue exhibits dense cellular architecture and lacks a clearly demarcated boundary from the surrounding normal brain parenchyma, indicating pronounced invasiveness (Figure S1 f&g). The tumor formed a well-defined intracranial mass. Within some tumor masses, distinct cavity-like structures with clear boundaries were observed, which we speculate represent intratumoral micro vessels. The tumor region exhibited marked cellular atypia and high cellular density (outlined by the white dashed line, figure S1g), a feature that was particularly evident in the DAPI-stained nuclei. As shown in Supplementary Figure S1H, tumor cells are distributed throughout the brain as satellite lesions or small cellular aggregates, consistent with the gross infiltrative growth pattern of the intracranial PCNSL model. Ki-67 immunostaining is performed on tumor sections from the HKBML and U2932 groups, as well as from human PCNSL specimens, to assess tumor proliferative activity. The HKBML group, representing CNS-derived PCNSL, shows slightly higher proliferation than the systemic DLBCL model (U2932 group) (Figure S1b). Notably, the Ki-67 index is highest in human PCNSL tissues (Figure S1d, S1e); however, the differences among the three groups do not reach statistical significance in Ki-67 mean intensity. Among Ki-67 mean area, there is statistical significance between U2932 group and HKBML group, however, human PCNSL tissue do not express statistical significance with HKBML group. This observation supports the survival data in vivo models, where the HKBML group exhibits a significantly poorer prognosis.

Together with prior single-cell transcriptomic analyses and our intracranial animal models, our data suggests that PCNSL exhibits highly aggressive biological behavior within the CNS microenvironment. Rather than indicating intrinsic biological differences between PCNSL and systemic DLBCL, our findings support the concept that the unique intracranial microenvironment may substantially influence tumor progression. Resident CNS immune cells, represented by microglia, together with local cytokine signaling and metabolic interactions, may contribute to tumor growth and immune evasion. These observations are consistent with the clinical characteristics of PCNSL and prompted us to focus on microglia, the principal immune effector cells in the CNS, to investigate their role in shaping the PCNSL tumor microenvironment.

Across all three groups, the distance regions consistently exhibited significantly lower Ki-67 expression than the core regions (Figure S1A). Nevertheless, quantitative analysis demonstrated that intracranial human PCNSL tissues retained relatively high proliferative activity even in distance regions (Figure S1C). These findings are consistent with the diffuse infiltrative growth pattern of PCNSL within the CNS, suggesting that regions distant from the tumor core may harbor infiltrating tumor cells or microscopic tumor foci that are not readily detectable by conventional histological assessment.

### Divergent microglia dynamic in PCNSL indicates distinct immune microenvironment

**Fig. 2:**
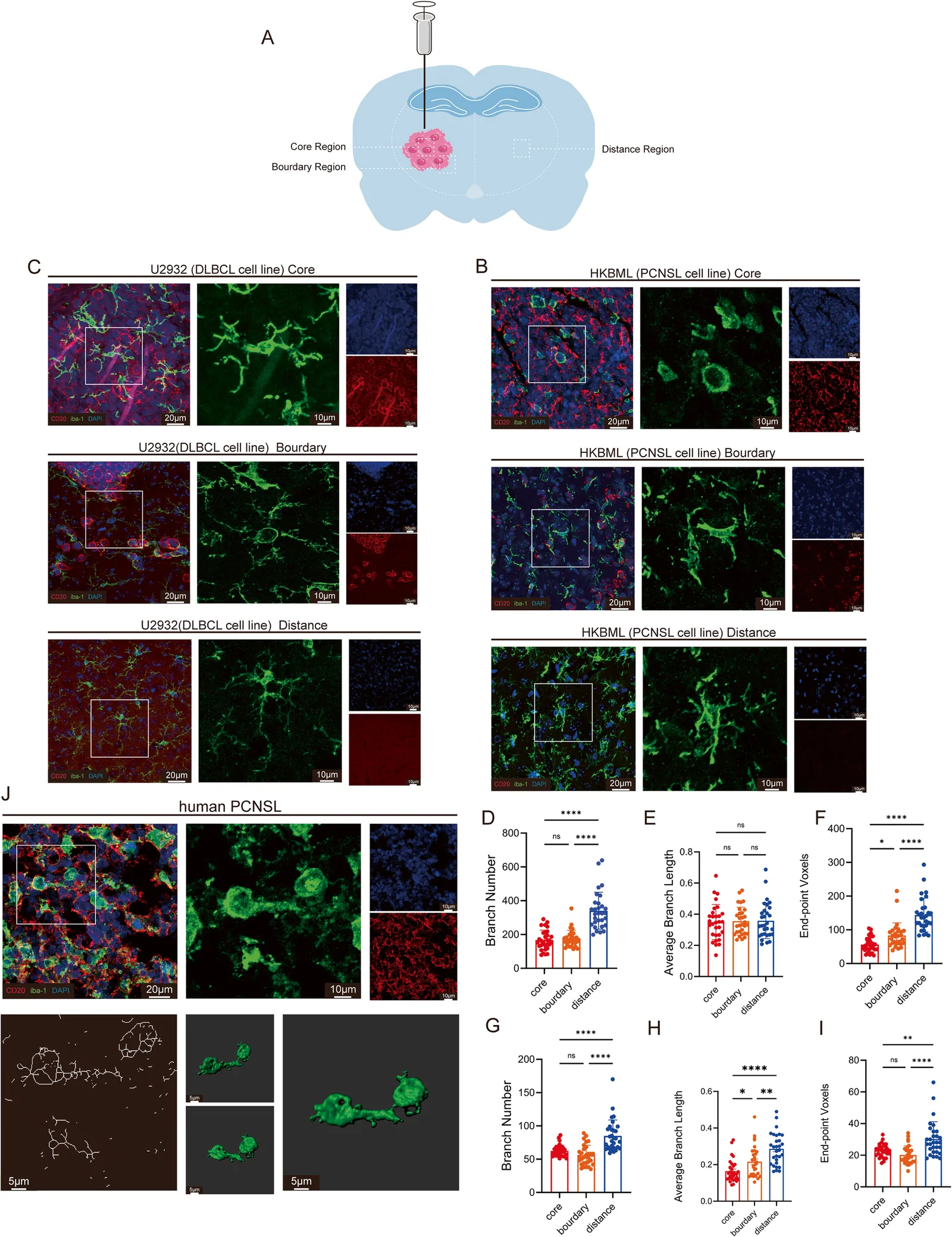
Divergent microglia dynamic in PCNSL indicates distinct immune microenvironment. **a** Schematic illustration of the tumor core region, tumor boundary region, and distant region. **b** Representative immunofluorescence images from the HKBML group showing microglia in the tumor core, boundary, and distal (non-infiltrated) regions. Scale bar, 20 μm. Insets display higher-magnification views of individual microglia within the indicated white boxes. Scale bar, 10 μm. **c** Representative immunofluorescence images from the U2932 group showing microglia in the tumor core, boundary, and distal (non-infiltrated) regions. Scale bar, 20 μm. Insets display higher-magnification views of individual microglia within the indicated white boxes. Scale bar, 10 μm. **d**-**f** Quantification of branch number, average branch length, and end-point voxels of microglia in the indicated regions of the U2932 group. Data are presented as mean ± SEM (*n* = 30 cells; 3 biological replicates, with 10 microglia randomly selected per image). **g-i** Quantification of branch number, average branch length, and end-point voxels of microglia in the indicated regions of the HKBML group. Data are presented as mean ± SEM (*n* = 30 cells; 3 biological replicates, with 10 microglia randomly selected per image). **j** Representative immunofluorescence images from human PCNSL tissue showing microglia in the tumor core, regions. Scale bar, 20 μm. Insets display higher-magnification views of individual microglia within the indicated white boxes. Scale bar, 10 μm. Skeletonization and 3D reconstruction of microglia highlighted in the white box in (j), Left: ImageJ-based skeletonization. Middle: 3D reconstruction shown in oblique (top) and lateral (bottom) views. Right: Frontal view of the reconstructed microglia. Scale bar, 5 μm. Statistical significance was determined using one-way ANOVA followed by Tukey’s multiple comparisons test. *P < 0.05, **P < 0.01, ***P < 0.001, ****P < 0.0001

Based on distinct cell lines derived from both systemic DLBCL and PCNSL, we establish two intracranial lymphoma models to investigate their biological behavior within the brain microenvironment (Fig. 2a). The core region was defined as the tumor core containing abundant CD20-positive lymphoma cells. The distance region was defined as an entire microscopic field located more than 2 mm away from the tumor mass, in which no detectable CD20-positive cells were observed. To minimize regional variation, the distance region was generally selected from the corresponding anatomical area in the contralateral hemisphere (Fig. 2a & Figure S2g). As described above, the HKBML group constitutes an authentic PCNSL model, derived from a patient with histopathologically confirmed PCNSL. In contrast, the U2932 group represents systemic ABC-DLBCL, which has been widely used in previous PCNSL modeling studies despite its peripheral origin. Given that both models share the same pathological subtype—ABC-DLBCL—we next focus on the differences in their tumor-associated immune microenvironments, particularly the behavior and activation status of microglia.

In the U2932 group, microglia located within the tumor core display a pronounced activated morphology, characterized by a reduced branch number, fewer end-point voxels, and simplified, retracted processes (Fig. 2c). This morphological change indicates a transition from the homeostatic, ramified state to an activated state commonly associated with inflammation or tissue injury. At the tumor margin, microglia also exhibit signs of activation, although to a lesser degree, with moderate reductions in branching complexity. In contrast, microglia in non-infiltrated brain regions maintain a classic resting morphology, with extensive branching, high branch number, and long average branch length.

Quantitative analysis of microglial morphology confirms significant regional differences. The branch number and end-point voxels show statistically significant reductions from normal tissue to tumor core (Fig. 2c and d), consistent with progressive activation. Interestingly, the average branch length does not differ significantly among the three regions (Fig. 2e), suggesting that while branching complexity is altered, process length remains relatively preserved in this model.

In the HKBML group, which represents the PCNSL model, microglia exhibit a markedly different pattern of activation. In the tumor core, microglia display a more extreme activated phenotype compared to the U2932 group, with substantial reductions in all three morphological parameters: branch number, average branch length, and end-point voxels (Fig. 2b). This suggests a more profound response to the tumor microenvironment. At the tumor margin, microglia are partially activated, while in the surrounding normal tissue, microglia retain a resting morphology, indicative of spatially restricted activation. Quantitative morphological analysis further supports this observation: branch number, average branch length, and end-point voxels are all significantly decreased in the tumor core compared to both the margin and normal regions (Fig. 2g–i).

These results collectively demonstrate that in both models, microglia within the tumor core adopt an activated phenotype, but the extent of activation is significantly greater in the HKBML group. A spatial gradient of activation is observed, with activation levels declining from the tumor core to the tumor margin and disappearing in the normal brain regions. This pattern reflects the ability of microglia to sense and respond to changes in the tumor microenvironment with high spatial precision.

To further validate these findings, we perform skeletonization and three-dimensional reconstruction of microglia in each region, confirming the progressive loss of branching and shift toward amoeboid morphology in tumor-infiltrated regions (Figure S2a–f). Notably, the microglial activation observed in the HKBML group is significantly more pronounced than that in U2932 group, suggesting that the CNS origin of the tumor may elicit a stronger local immune response.

To explore whether these features are also observed in clinical specimens, we perform Iba1 immunofluorescence on patient-derived PCNSL tissue. Consistent with our PCNSL model findings, microglia in patient tumors display an activated morphology, with markedly reduced branch number and end-point voxels (Fig. 2j). Sectional analysis and 3D reconstruction confirm the presence of highly activated, spherical-shaped microglia with minimal branching, resembling the microglial profile observed in the HKBML intracranial model. These observations reinforce the translational relevance of our PCNSL model and highlight the unique immunological features of the CNS lymphoma microenvironment.

### Microglial subsets exhibit unique immunosuppressive signatures in the PCNSL CNS microenvironment

**Fig. 3:**
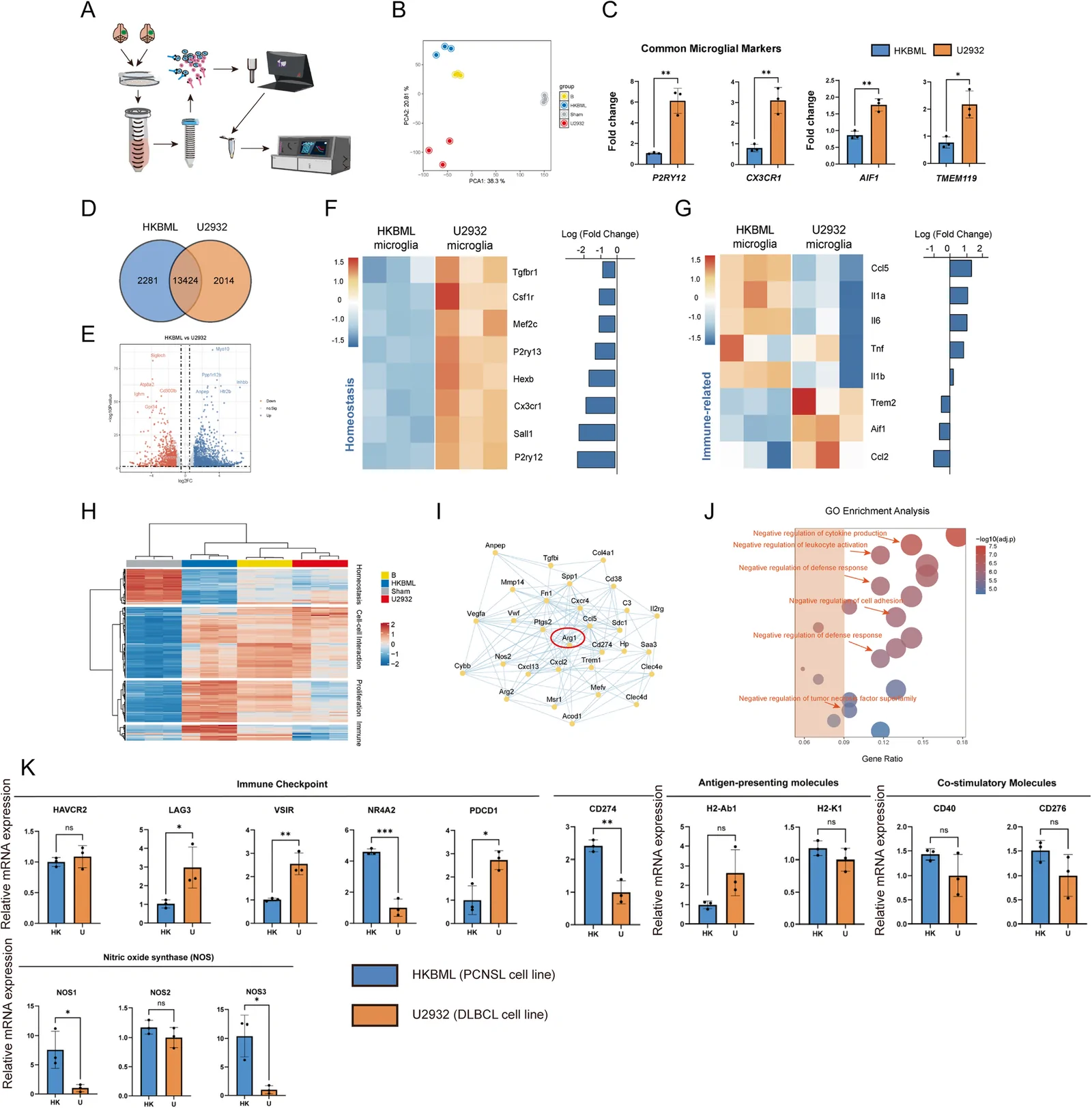
Transcriptomic profiling of microglia reveals distinct immunological signatures in PCNSL. **a** Schematic illustration of magnetic-activated cell sorting (MACS) used to isolate CD11b⁺ microglia from mouse brain tissue. **b** Principal component analysis (PCA) demonstrates distinct transcriptomic profiles among microglia from HKBML and U2932 groups. **c** Expression levels of canonical microglial markers (P2RY12, CX3CR1, AIF1, TMEM119) across groups. **d** Venn diagram showing the number of shared and unique differentially expressed genes (DEGs) between HKBML and U2932 groups. **e** Volcano plot of microglia RNA-seq data comparing HKBML and U2932 groups. **f** Comparative expression of microglial homeostasis-associated genes between HKBML and U2932 groups. **g** Differential expression of immune-related genes in microglia from HKBML and U2932 groups. **h** Unsupervised clustering identifies a disease-associated microglial subpopulation enriched in ARG1 expression. **i** Protein–protein interaction (PPI) network analysis of key genes within the ARG1⁺ microglial subset. **j** Gene Ontology (GO) enrichment analysis of the ARG1⁺ microglial subpopulation reveals involvement in immunosuppressive and metabolic pathways. **k** Expression of immune suppression‑related markers in microglia

To further investigate the role of microglia in PCNSL specific TME, microglia are isolated from 8-week-old mice in the Sham, HKBML, U2932, and resveratrol groups using magnetic-activated cell sorting (Fig. 3a), followed by RNA sequencing (RNA-seq). Principal component analysis (PCA) reveals distinct transcriptomic profiles among the four groups (Fig. 3b). Comparative analysis between the HKBML and U2932 groups shows that classical microglial markers (P2ry12, Cx3cr1, Aif1, and Tmem119) are more highly expressed in the U2932 group (Fig. 3c). Differential gene expression analysis identifies 2,014 upregulated and 2,281 downregulated genes in U2932 microglia relative to those in the HKBML group (Fig. 3d), as illustrated in the volcano plot (Fig. 3e). U2932-derived microglia exhibit upregulation of homeostasis-associated genes such as Csf1r, Mef2c, P2ry13, and Hexb (Fig. 3f). Conversely, microglia in the HKBML group demonstrate elevated expression of immune-related genes including Ccl5, Il1b, Il1a, and Tnf, while Trem2, Aif1, and Ccl2 are preferentially expressed in U2932 (Fig. 3g). Unsupervised clustering categorizes differentially expressed genes into four functional clusters: homeostasis, cell-cell interaction, proliferation, and immune-related pathways. Notably, immune-related genes show marked enrichment in the HKBML group, suggesting a distinct immune activation profile compared to U2932 (Fig. 3h). Protein-protein interaction (PPI) analysis identifies Arg1 as a central hub gene within the immune-related network (Fig. 3i). Gene Ontology (GO) enrichment analysis further indicates that these immune genes are primarily associated with immunosuppressive processes, suggesting that microglia in the HKBML group may contribute to a more immunosuppressive tumor microenvironment. This immunosuppressive signature potentially accounts for the shorter survival observed in HKBML tumor-bearing mice (Fig. 3j). To further characterize the immunoregulatory features of PCNSL-associated microglia, we examined the expression of genes involved in immune checkpoint signaling, antigen presentation, and arginine metabolism. Among the immune checkpoint-related genes, NR4A2 was significantly upregulated, whereas LAG3 was downregulated in HKBML-associated microglia compared with the U2932 group. In contrast, HAVCR2 (TIM-3), VSIR (VISTA), PDCD1 (PD-1), and CD274 (PD-L1) showed no significant differences between the two groups. Likewise, the antigen presentation and co-stimulatory molecules H2-Ab1, H2-K1, CD40, and CD276 were comparably expressed. Interestingly, analysis of the nitric oxide synthase family revealed significantly increased expression of NOS1 and NOS3, whereas NOS2 remained unchanged in PCNSL-associated microglia. Together with the elevated expression of ARG1, these findings suggest that metabolic reprogramming, rather than broad activation of classical immune checkpoint pathways or antigen presentation programs, may represent a characteristic feature of PCNSL-associated microglia.

### ARG1⁺ microglial subpopulation shapes the immune landscape of PCNSL by secrete cytokines

Transcriptomic profiling of microglia isolated from the HKBML group reveals a distinct subpopulation of microglia characterized by dysregulated arginine metabolism. Among the differentially expressed genes, protein–protein interaction (PPI) network analysis identifies ARG1 (arginase 1) as a core hub gene, showing significant upregulation in this subpopulation. These cells display gene signatures consistent with disease-associated microglia (DAM), a phenotype known to emerge in response to pathological cues in PCNSL TME.

**Fig. 4:**
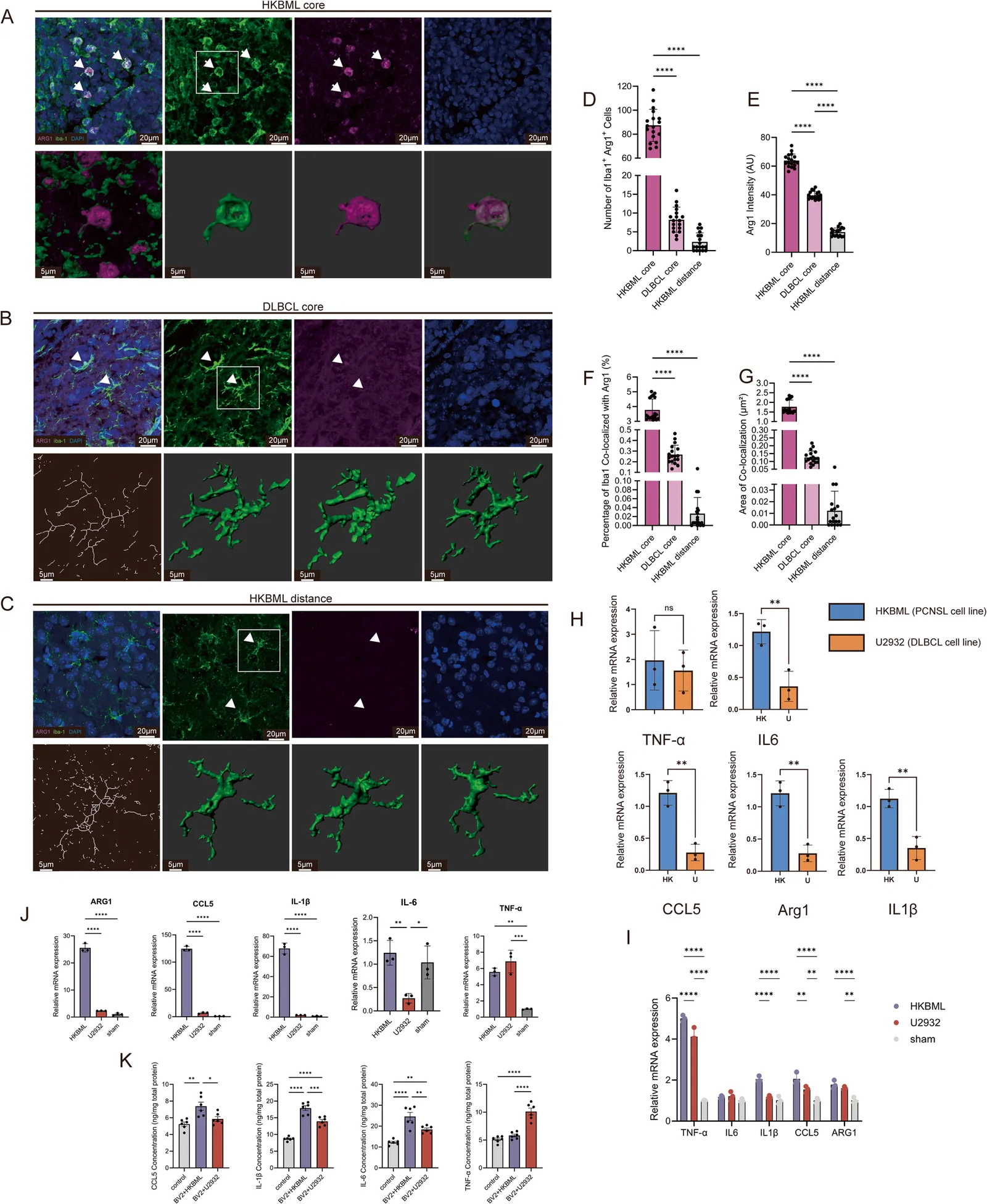
A Distinct ARG1⁺ Microglial Subpopulation Defines the Immune Landscape of PCNSL. **a** Representative immunofluorescence images from the HKBML group showing microglia in the tumor core region. Scale bar, 20 μm. White arrows indicate cells co-expressing Iba1 and ARG1. The microglia highlighted within the white box are shown in the second row for 3D reconstruction. Scale bar, 5 μm. **b** Representative immunofluorescence images from the DLBCL group showing microglia in the tumor core region. Scale bar, 20 μm. White arrowheads denote Iba1⁺ microglia lacking ARG1 co-labeling. The microglia highlighted within the white box are shown in the second row for skeletonization and 3D reconstruction. **c** Representative immunofluorescence images from the HKBML group showing microglia in the distance region. Scale bar, 20 μm. White arrowheads denote Iba1⁺ microglia lacking ARG1 co-labeling. The microglia highlighted within the white box are shown in the second row for skeletonization and 3D reconstruction. **d**-**g** Quantification of number of iba1+ARG1+ cells (**d**), ARG1 intensity (**e**), percentage of iba1+ co-localized ARG1+ cells (**f**), area of co-localization (**g**) in the indicated regions of above groups. Data are presented as mean ± SEM (*n* = 6 slides per group; 3 independent experiments). **h** Transcriptomic comparison of immune-related gene expression between HKBML and U2932 groups. Data are presented as mean ± SEM (*n* = 3 biological replicates per group). Statistical analysis is performed using one-way ANOVA followed by Tukey’s post hoc test. **i** Quantitative PCR analysis of immune-related gene expression in microglia. Bars represent mean ± SEM (*n* = 3 biological replicates). **j** Quantitative PCR analysis of immune-related gene expression in tumor tissues. Bars represent mean ± SEM (*n* = 3 biological replicates). Statistical significance is determined using unpaired two-tailed Student’s t test. **k** Quantification of cytokine levels in the cell co-culture model *P < 0.05, **P < 0.01, ***P < 0.001, ****P < 0.0001

To validate the presence of this ARG1⁺ disease-associated microglial subpopulation in vivo, we perform dual immunofluorescence staining for Iba1 and ARG1. In the tumor core region of the HKBML group (PCNSL), numerous highly activated microglia are observed, as previously described. Notably, these Iba1⁺ microglia exhibit strong intracellular expression of ARG1, supporting their identity as a distinct ARG1⁺ DAM enriched in the PCNSL microenvironment. Three-dimensional reconstruction further confirms widespread co-expression of Iba1 and ARG1 among tumor-infiltrating microglia (Fig. 4a).

In contrast, within the core region of the U2932 (systemic DLBCL) model, microglia display an amoeboid activated dynamics with reduced branch number and shortened average branch length (Fig. 4b), but ARG1 expression is largely absent. Co-localization of ARG1 with Iba1 is rarely detected, suggesting that the ARG1⁺ microglial subset is not a prominent feature in systemic DLBCL. Similarly, in the non-infiltrated brain regions of the HKBML group, microglia retain a homeostatic morphology and show minimal to no ARG1 expression (Fig. 4c), further indicating that ARG1⁺ microglia are spatially restricted to the tumor microenvironment.

Quantitative analysis supports these findings. In the tumor core region of the HKBML model, both the absolute number and proportion of ARG1⁺&Iba1⁺ microglia are significantly elevated compared to the tumor core region of the U2932 group and to normal brain regions (Fig. 4d and f). In contrast, ARG1 fluorescence intensity and the co-localized area with Iba1 are markedly reduced in non-infiltrated regions of the HKBML group (Fig. 4e and g). Based on transcriptomic data, we select several immune-related molecules for validation (Fig. 4h). Using a cell co-culture model, we find that immunosuppressive genes in microglia co-cultured with HKBML are significantly upregulated, consistent with the sequencing results (Fig. 4i). We perform qPCR to examine the expression levels of several immunosuppressive genes, including CCL5, IL-1β, IL-6, and ARG1, in tumor tissues. Consistent with the results observed in the cellular model, the expression of these immunosuppressive genes is upregulated in vivo (Fig. 4j). To further characterize the functional phenotype of microglia, we quantified cytokine secretion in the microglia–tumor cell co-culture system. Compared with the DLBCL co-culture group, microglia co-cultured with PCNSL cells secreted significantly higher levels of the immunosuppressive cytokines IL-1β and IL-6, while exhibiting reduced secretion of the pro-inflammatory cytokines TNF-α and IFN-γ, indicating a shift toward an immunosuppressive phenotype (Fig. 4k).

To further characterize microglial activation in different tumor models, we performed multiplex immunofluorescence staining for the M1 marker iNOS and the M2 marker CD206 in the HKBML and DLBCL groups. In the HKBML tumor core, microglia displayed activated morphological features, including reduced branching and shortened processes (Figure S5a&b). iNOS fluorescence intensity was significantly increased in the tumor core compared with distant regions in both HKBML and DLBCL groups, with no significant difference between the two models (Figure S5c–f). In contrast, CD206 expression was markedly higher in the HKBML tumor core than in the DLBCL group, while remaining low in distant regions in both models (Figure S6).

To further validate our findings in vitro, we established a co-culture model using HKBML tumor cells and microglia. In this system, microglia exhibited significantly increased fluorescence intensity of CD206, iNOS, and ARG1 compared with control groups (Figure S7a-c). Notably, the concurrent upregulation of both M1- and M2-associated markers does not indicate classical polarization toward a specific subtype but rather reflects a robust activation state of microglia (Figure S7d). Upon treatment with an ARG1 inhibitor, the fluorescence intensities of these markers were markedly reduced, suggesting that ARG1 inhibition attenuates microglial activation. To further elucidate the underlying mechanisms, microglia were isolated by magnetic bead sorting and subjected to transcriptomic analysis. Gene Ontology (GO) enrichment analysis revealed significant enrichment in pathways related to cell migration and cytoskeletal organization, indicating dynamic structural remodeling of activated microglia (Figure S7f). Transcriptomic analysis and qPCR validation of isolated microglia further revealed enrichment of pathways associated with cell migration, cytoskeletal remodeling, and PI3K–AKT signaling (Figure S7e-h). These findings suggest that ARG1 upregulation disrupts microglial homeostasis, leading to activation-associated morphological changes, including alterations in branching and synaptic interactions. Moreover, activation of the PI3K–AKT pathway may promote the secretion of immunosuppressive cytokines, thereby contributing to tumor microenvironment remodeling.

These findings indicate that microglia in the HKBML tumor core preferentially acquire an M2-like immunosuppressive phenotype. Together, these results support the notion that the HKBML microenvironment drives a skewing of activated microglia toward an M2-polarized state, further contributing to the immunosuppressive landscape of PCNSL. Collectively ARG1⁺ disease-associated microglia constitute a spatially confined and PCNSL-specific subpopulation, which may contribute to the immunometabolic remodeling of the tumor microenvironment.

### Resveratrol remodels microglia dynamics and suppresses tumor progression in PCNSL

In our research, we screened 31 newly isolated small-molecule compounds derived from a secondary metabolite of Ganoderma lucidum, a traditional medicinal fungus known for its anti-inflammatory and immunomodulatory properties (Figure S3a, S3b). The chemical structures of these compounds were determined by high-resolution mass spectrometry, and full characterization data are available in the Supplementary Materials.

**Fig. 5:**
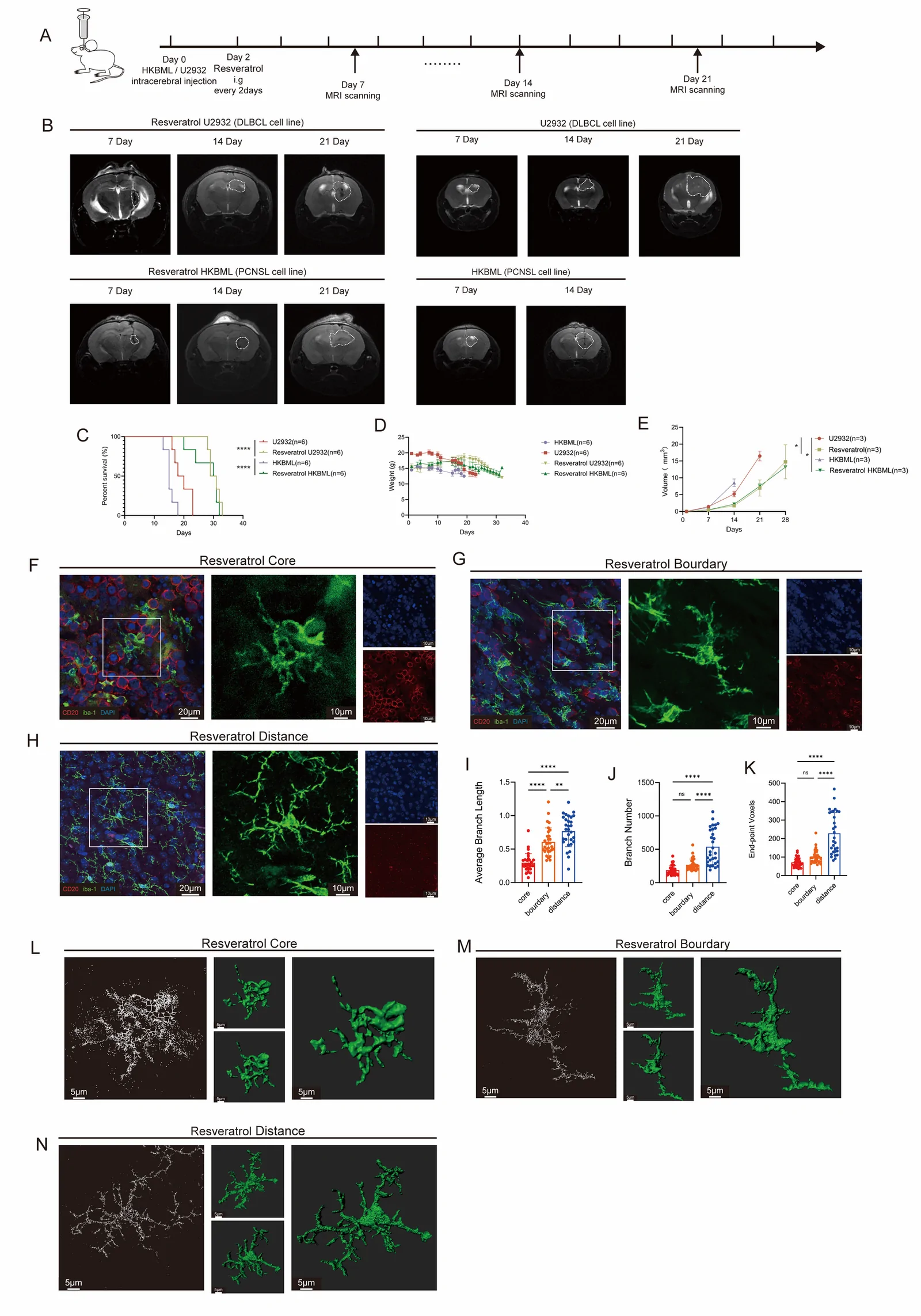
Resveratrol remodels microglia dynamics and suppresses tumor progression in PCNSL. **a** Schematic diagram of the PCNSL mice model treated with resveratrol. **b** Representative images of 7.0T MRI images of mice brains were acquired at days 7, 14, and 21 post-implantation with resveratrol-treated HKBML group or U2932 group, area within the white dotted line is tumor region. **c** Kaplan–Meier survival curves of HKBML group and U2932 group with or without resveratrol treatment in vivo (Data represented as mean ± SEM, *n* = 6). **d** Body weight changes in groups mentioned above (Data represented as mean ± SEM, *n* = 6). **e** Quantification of MRI scanning tumor volume of HKBML group and U2932 group with or without resveratrol treatment(Data represented as mean ± SEM, *n* = 3). **f**-**h** Representative immunofluorescence images from the resveratrol-treated U2932 group showing microglia in the tumor core, boundary, and distal (non-infiltrated) regions. Scale bar, 20 μm. Insets show microglia at higher magnification as indicated by the area in white box. Scale bar, 10 μm. **i-k** Quantification of branch number (**j**), average branch length (**i**), and end-point voxels (**k**) of microglia in the indicated regions of the resveratrol-treated U2932 group. Data are presented as mean ± SEM (*n* = 30 cells; 3 biological replicates, with 10 microglia randomly selected per image). **l-n**, Representative image of 3D reconstruction and skeletonization in different regions in resveratrol-treated U2932 group. Middle: 3D reconstruction shown in oblique (top) and lateral (bottom) views. Right: Frontal view of the reconstructed microglia. Scale bar, 5 μm. Statistical significance was determined using one-way ANOVA followed by Tukey’s multiple comparisons test. *P < 0.05, **P < 0.01, ***P < 0.001, ****P < 0.0001

Dual in vitro and in vivo screening using HKBML and U2932 intracranial tumor models led to the identification of a lead candidate compound, resveratrol (Fig. 5a). In intracranial tumor models, resveratrol treatment significantly suppressed intracranial tumor growth and improved overall survival in both models (Fig. 5b). Specifically, in the U2932 group, median survival was extended from 19 to 29 days, while in the HKBML group, resveratrol prolonged median survival from 14 to 30 days (Fig. 5c). Body weight curves also supported these findings, demonstrating delayed onset of cachexia and improved physical condition in resveratrol-treated mice (Fig. 5d). Tumor growth kinetics were further assessed using high-resolution 7.0T magnetic resonance imaging (MRI) at serial time points. In both HKBML group and U2932 group, mice treated with resveratrol exhibited significantly smaller tumor volumes on days 14 and 21 compared to untreated controls (Fig. 5e). No significant difference was observed on day 7, suggesting that resveratrol delays rather than prevents early tumor establishment. Notably, tumor volumes in the resveratrol group reached approximately 8 mm³ by day 21, a size that was already observed in the control group as early as day 14, indicating a temporal delay in tumor progression. A significant reduction in tumor volume measured by MRI was observed in the DLBCL group following resveratrol treatment, whereas no significant difference was detected between the HKBML group and the corresponding treatment group. We speculate that this is attributable to the limited survival of mice bearing HKBML tumors, as most animals did not survive until the third MRI examination (day 21), precluding reliable assessment of tumor volume at this time point. Nevertheless, both the survival curves and body weight changes consistently indicated a trend toward greater malignancy in the HKBML intracranial model. To evaluate the impact of resveratrol on the tumor microenvironment, particularly on microglia—the key immune effector cells in the CNS—we performed histological and 3D morphological analyses of tumor-associated microglia (Fig. 5f-h). In the tumor core of resveratrol-treated mice, microglia displayed activated morphologies characterized by reduced branch number, shortened average branch length, and a decreased number of end-point voxels, indicative of functional activation (Fig. 5i-k). These changes were confirmed by three-dimensional reconstruction and skeletonization analysis, supporting the notion that resveratrol induces spatial remodeling of microglial structure and function within the tumor niche (Fig. 5l-m). Interestingly, although resveratrol exerts significant antitumor effects, it does not suppress microglial activation; rather, it appears to reshape their activation dynamics. This suggests that resveratrol may not simply act by eliminating tumor burden but may also reshape the TME of PCNSL. Whether resveratrol exerts a direct effect on microglia or whether microglial remodeling is secondary to tumor reduction remains to be determined. To further investigate the direct immunomodulatory effects of resveratrol on microglia, cytokine secretion was quantitatively analyzed under both monoculture and microglia–tumor cell co-culture conditions. In microglial monoculture, resveratrol significantly increased the secretion of the antitumor cytokines TNF-α and IFN-γ, suggesting that resveratrol directly activates microglia and promotes their polarization toward an antitumor phenotype. In the microglia–tumor cell co-culture system, resveratrol not only enhanced the production of TNF-α and IFN-γ but also markedly reduced the elevated levels of the tumor-promoting cytokines IL-1β and IL-6 induced by co-culture, indicating that resveratrol effectively reverses the immunosuppressive cytokine profile of PCNSL-associated microglia (Figure S7i).

### Resveratrol targets ARG1⁺ disease-associated microglia to remodel the tumor microenvironment and inhibit PCNSL growth

Tumor-bearing mice in the HKBML group were treated with resveratrol via oral gavage, and brain tissues were harvested post-mortem for immunofluorescence analysis (Fig. 5a).

**Fig. 6:**
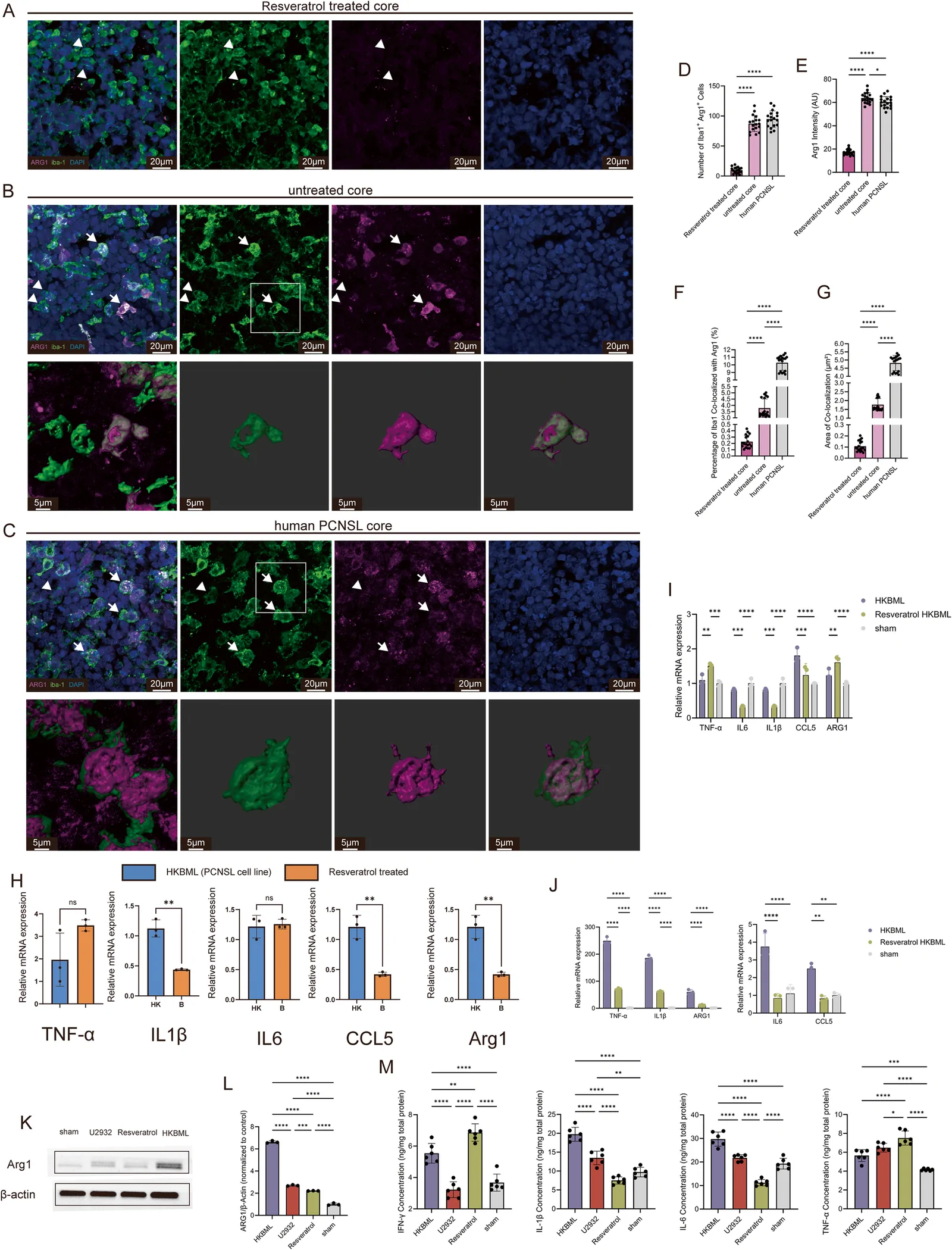
Resveratrol Targets ARG1⁺ Disease-Associated Microglia to Remodel the Tumor Microenvironment. **a** Representative immunofluorescence images from the resveratrol-treated HKBML group showing microglia in the tumor core region. Scale bar, 20 μm. White arrowheads denote Iba1⁺ microglia lacking ARG1 co-labeling. **b** Representative immunofluorescence images from the untreated HKBML group showing microglia in the tumor core region. Scale bar, 20 μm. White arrows indicate cells co-expressing Iba1 and ARG1. White arrowheads denote Iba1⁺ microglia lacking ARG1 co-labeling. The microglia highlighted within the white box are shown in the second row for 3D reconstruction. Scale bar, 5 μm. **c** Representative immunofluorescence images from human PCNSL tissue showing microglia in the tumor core region. Scale bar, 20 μm. White arrows indicate cells co-expressing Iba1 and ARG1. White arrowheads denote Iba1⁺ microglia lacking ARG1 co-labeling. The microglia highlighted within the white box are shown in the second row for 3D reconstruction. Scale bar, 5 μm. **d-g** Quantification of number of iba1+ARG1+ cells (**d**), ARG1 intensity (**e**), percentage of iba1+ co-localized ARG1+ cells (**f**), area of co-localization in the indicated regions (**g**) of above groups. Data are presented as mean ± SEM (*n* = 6 slides per group; 3 independent experiments). **h** Transcriptomic comparison of immune-related gene expression between HKBML and resveratrol-treated groups. Data are presented as mean ± SEM (*n* = 3 biological replicates per group). Statistical analysis is performed using one-way ANOVA followed by Tukey’s post hoc test. **i** Quantitative PCR analysis of immune-related gene expression in microglia. Bars represent mean ± SEM (*n* = 3 biological replicates). **j** Quantitative PCR analysis of immune-related gene expression in tumor tissues. Bars represent mean ± SEM (*n* = 3 biological replicates). **k**,** l** Representative western blot and quantitative analysis of ARG1 expression in tumor tissues. β-actin was used as a loading control. Data are presented as mean ± SEM (*n* = 3 biological replicates per group). **m** Quantitative analysis of cytokine levels in tumor tissues measured by ELISA. Data are presented as mean ± SEM (*n* = 6 biological replicates per group). Statistical significance was determined using one-way ANOVA followed by Tukey’s multiple comparisons test. *P < 0.05, **P < 0.01, ***P < 0.001, ****P < 0.0001

Co-staining for Iba1 and ARG1 revealed a striking reduction in ARG1 expression within the tumor core following resveratrol treatment (Fig. 6a). Notably, co-localization between Iba1⁺ microglia and ARG1 was almost completely abolished in the treated group. In contrast, the untreated HKBML group retained a dense population of ARG1⁺&Iba1⁺ microglia within the tumor core, consistent with our prior observations (Fig. 6b). Three-dimensional reconstruction further confirmed the spatial separation of microglia from ARG1 expression after resveratrol exposure, suggesting a disruption or reversal of the ARG1⁺ disease-associated microglial phenotype.

Similar patterns were observed in human PCNSL specimens (Fig. 6c), where a large number of Iba1⁺ microglia co-expressed high levels of ARG1 within the tumor core, reinforcing the translational relevance of this phenotype. Quantitative fluorescence analysis supports these findings: resveratrol treatment led to a significant reduction in ARG1 signal intensity within the tumor core, as well as a marked decrease in both the absolute number and percentage of ARG1⁺Iba1⁺ double-positive cells (Fig. 6d-g).

In contrast, untreated HKBML tumors and patient-derived specimens exhibited extensive ARG1⁺ microglial infiltration. Moreover, quantitative comparisons showed that the number, proportion, and total co-localized area of ARG1⁺Iba1⁺ cells were significantly higher in human PCNSL specimens than in the HKBML group (Fig. 6d-g). After resveratrol treatment in the cell co-culture model, immune-related molecules in microglia show improvement, including a significant increase in TNF-α and downregulation of immunosuppressive factors such as IL-6 and CCL5 (Fig. 6h and i). Interestingly, however, the mRNA level of ARG1 increases after resveratrol treatment, which contrasts with our sequencing results. We next evaluate the overall immunosuppressive status of the tumor microenvironment using tumor tissues and find that resveratrol suppresses the expression of immunosuppressive cytokines such as CCL5, IL-6, IL-1β, and ARG1, although TNF-α is also reduced (Fig. 6j). We then use ELISA to assess the protein levels of IFN-γ, IL-6, TNF-α, and IL-1β (Fig. 6m). The results show that resveratrol treatment increases the levels of the antitumor cytokines IFN-γ and TNF-α, while decreasing IL-6 and IL-1β levels. Notably, the HKBML group exhibits lower expression of antitumor cytokines (IFN-γ and TNF-α) but higher levels of immunosuppressive cytokines compared with the U2932 group. Finally, western blot analysis confirms that ARG1 expression, a key immunoregulatory molecule, is reduced following resveratrol treatment (Fig. 6k and l). The suppression of ARG1 by resveratrol observed in vivo through immunofluorescence likely reflects a more physiologically relevant response. Additionally, Resveratrol-mediated inhibition of ARG1 in microglia may trigger a compensatory transcriptional upregulation, resulting in the elevated mRNA levels detected by qPCR. To further determine whether resveratrol affects microglial infiltration or phenotype, we quantified the total number of Iba1⁺ microglia and the proportion of ARG1⁺Iba1⁺ cells (Figure S8). Resveratrol treatment did not significantly alter the overall number of infiltrating Iba1⁺ microglia. However, the proportion of ARG1⁺ microglia within the Iba1⁺ population was markedly reduced following treatment. Furthermore, both the total number of microglia and the proportion of ARG1⁺Iba1⁺ cells showed a progressive decline from the HKBML tumor core to the DLBCL tumor core and finally to the control regions. These findings indicate that ARG1⁺ microglia are preferentially enriched within the PCNSL tumor core and that resveratrol primarily suppresses their immunosuppressive phenotype rather than reducing overall microglial infiltration.

### Biosafety of resveratrol in vivo

**Fig. 7:**
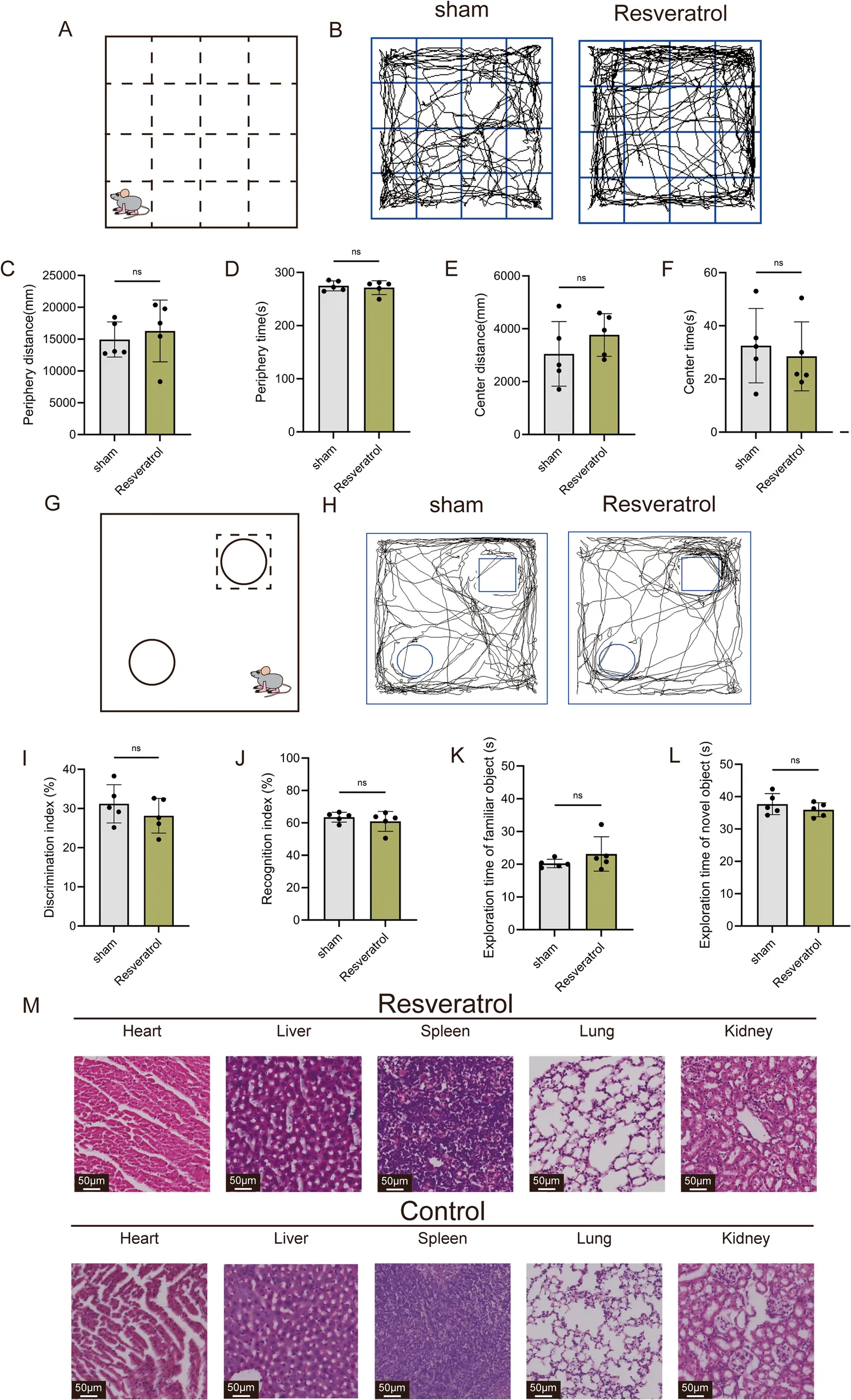
Biosafety of resveratrol in vivo. **a** Schematic diagram of the open field test showing the central and peripheral zones used for behavioral analysis. **b** Representative locomotor trajectories of mice in the open field test from the control group and resveratrol-treated group. **c-f** Quantification of behavioral parameters in the open field test, **c** total distance traveled in the central zone, **d** total distance in the peripheral zone, **e** time spent in the central zone, and **f** time spent in the peripheral zone. Data are presented as mean ± SEM (*n* = 5). Statistical significance was determined using unpaired two-tailed Student’s t-test. **g** Schematic diagram of the novel object recognition test showing the locations of familiar and novel objects used for behavioral analysis. **h** Representative locomotor trajectories of mice in novel object recognition test from the control group and resveratrol-treated group. **i**-**l** Quantification of behavioral parameters in the novel object recognition test: **i** discrimination index (DI), **j** recognition index (RI), **k** exploration time of the familiar object, and **l** exploration time of the novel object. Data are presented as mean ± SEM (*n* = 5). **m**, Representative H&E-stained sections of major organs (heart, liver, spleen, lung, and kidney) from control and resveratrol-treated mice, showing no observable histopathological abnormalities. Scale bar, 50 μm. *P < 0.05, **P < 0.01, ***P < 0.001, ****P < 0.0001

To evaluate the safety of resveratrol, behavioral assays and histopathological analyses were performed in healthy mice treated with therapeutic doses of resveratrol (Fig. 7a). Resveratrol was administered by oral gavage (i.g.) at a concentration of 1 mg/mL in normal saline, with each mouse receiving 0.2 mL per administration. In the open field test, resveratrol-treated mice showed no significant changes in locomotor activity or anxiety-like behavior compared with controls (Fig. 7b–f). Similarly, in the novel object recognition test, no significant differences were observed in recognition memory or exploratory behavior between the two groups, indicating that resveratrol did not impair cognitive function (Fig. 7g–l).

To further assess systemic toxicity, major organs were collected for histological examination. H&E staining revealed no obvious pathological abnormalities, inflammatory infiltration, or structural damage in the heart, liver, spleen, lungs, or kidneys of resveratrol-treated mice compared with controls (Fig. 7m). These findings demonstrate that resveratrol exhibits minimal neurobehavioral and systemic toxicity at therapeutic doses.

## Discussion

**Fig. 8:**
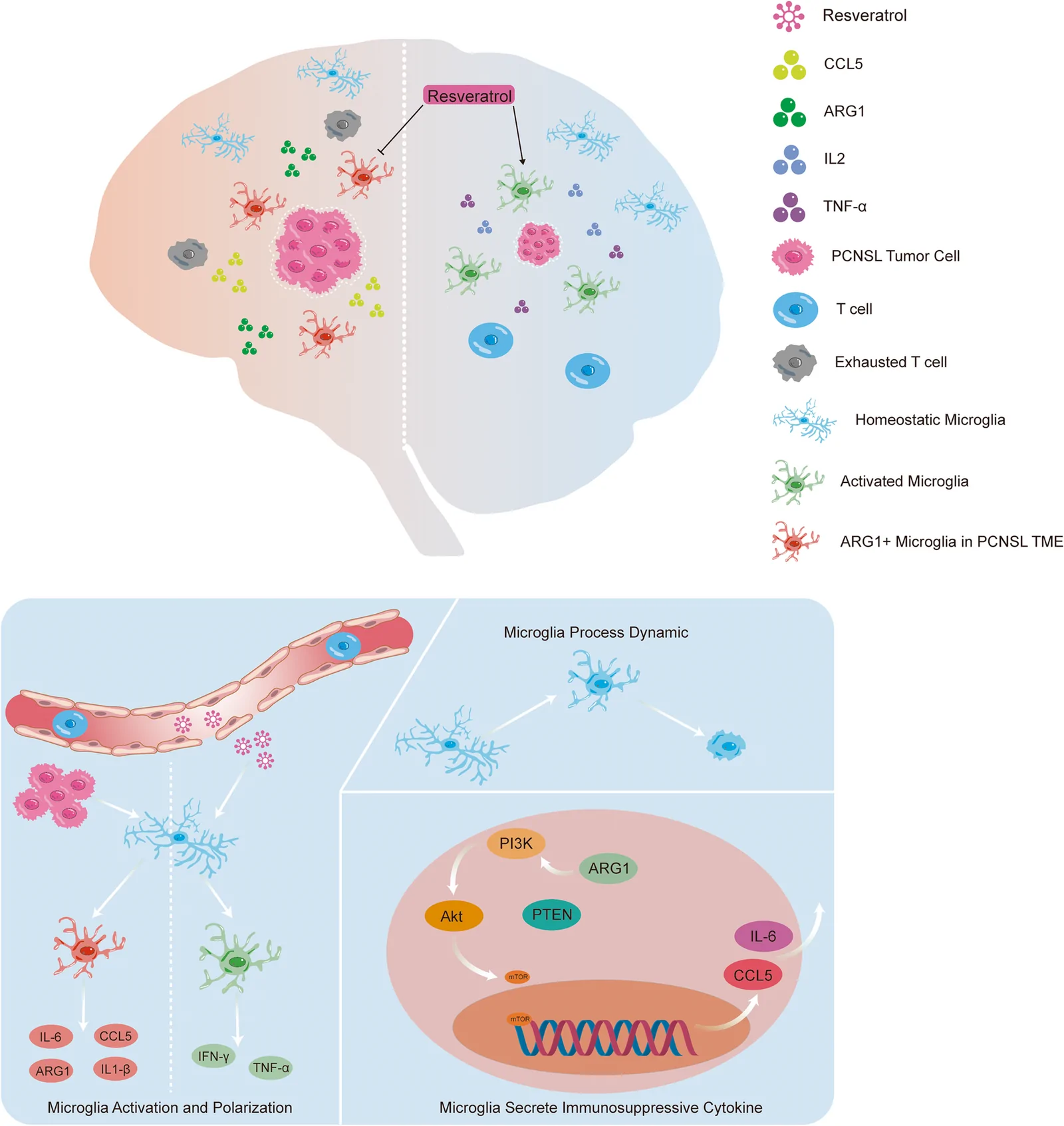
Schematic illustration of the microglia function in immunosuppressive tumor microenvironment in PCNSL and the regulatory effect of resveratrol

A defining hallmark of PCNSL is its localization within the central nervous system, a site historically considered immune-privileged. Recent discoveries, however, have redefined this notion, highlighting the critical role of microglia—the brain’s resident immune cells—in mediating immune surveillance, phagocytosis, and cytokine signaling [17, 26, 27]. Unlike secondary CNS lymphoma (SCNSL) [28], which represents metastatic spread of systemic disease, PCNSL typically originates and remains confined to the CNS, suggesting that the intracranial microenvironment plays a pivotal role in its pathogenesis. Our findings support this view: we identified a population of microglia within PCNSL lesions characterized by high expression of ARG1, consistent with a disease-associated microglial (DAM) phenotype. Notably, this phenotype is absent in systemic DLBCL cells, further emphasizing the role of the CNS milieu in shaping tumor–microglia interactions. These ARG1⁺ microglia exhibit a paradoxical activation state—morphologically activated yet functionally immunosuppressive—marked by reduced phagocytic capacity and increased expression of anti-inflammatory mediators that likely contribute to tumor evasion. This phenomenon underscores that microglial activation in PCNSL is not merely reactive but tumor-promoting, potentially forming a key component of immunosuppressive TME. Taking together, these data strongly argue that PCNSL is not simply a localized manifestation of systemic DLBCL, but rather a CNS-specific malignancy, shaped by and dependent on the unique features of the intracranial microenvironment. (Fig. 8).

In light of TME in PCNSL, particularly the presence of an immunosuppressive ARG1⁺ microglial subpopulation, our study explored a therapeutic approach that targets microenvironmental reprogramming rather than tumor cells alone. We identified a small-molecule compound, resveratrol, derived from Ganoderma lucidum, which effectively inhibits ARG1⁺ microglia both in vitro and in vivo. This intervention attenuated the immunosuppressive landscape of the PCNSL TME and led to significant tumor growth suppression and prolonged survival in preclinical models. Notably, resveratrol treatment resulted in a marked reduction in the co-expression of IBA1 and ARG1, supporting its potential role in modulating DAM toward a less tumor-permissive state. Consistent with recent studies, our findings suggest that microglia in the PCNSL microenvironment cannot be adequately classified using the traditional M1/M2 polarization framework. Instead, their phenotypic and functional heterogeneity is better characterized by distinct molecular programs, such as the ARG1-associated microglial state identified in this study. Nevertheless, the precise functional identity and regulatory mechanisms of this microglial population require further investigation.

An apparent difference was observed between ARG1 mRNA expression in the in vitro co-culture system and ARG1 protein expression in the in vivo model following resveratrol treatment. Although the present study was not designed to investigate the underlying regulatory mechanism, this discrepancy may reflect the complex regulation of ARG1 at multiple levels, including post-transcriptional and post-translational processes, as well as differences between the simplified in vitro co-culture system and the intracranial tumor microenvironment. In addition, temporal differences in sample collection may also contribute to these observations. Future studies combining matched analyses of ARG1 mRNA and protein expression from the same biological samples, together with mechanistic investigations, will be important to clarify the regulation of ARG1 during microglial reprogramming in PCNSL. Another limitation of this study is that ARG1 protein expression was validated by Western blot using tumor-containing brain tissues rather than magnetically isolated microglia. Although immunofluorescence co-localization, CD11b⁺ microglial RNA sequencing, and qPCR analyses consistently supported microglia as the major source of ARG1 expression, future studies using purified microglia for protein-level validation will further strengthen these findings.

Compared with methotrexate, which remains the backbone of PCNSL treatment despite well-known systemic toxicities—particularly nephrotoxicity—resveratrol demonstrated a favorable safety profile. Suggesting that resveratrol may offer a safer alternative or adjunct to current therapies, especially for patients with MTX intolerance.

Our study primarily focused on the reprogramming of microglia within the PCNSL microenvironment. Emerging evidence suggests that innate immune cells, particularly microglia, play a critical role in shaping adaptive immune responses. It is therefore plausible that resveratrol-induced microglial remodeling may also influence T-cell infiltration, differentiation, and functional states, including CD4⁺/CD8⁺ balance, regulatory T-cell abundance, and immune checkpoint expression. However, comprehensive flow cytometric profiling of tumor-infiltrating lymphocytes was not performed in the present study. Future studies incorporating multi-dimensional immune phenotyping will be necessary to fully elucidate the impact of microglial reprogramming on the broader tumor immune ecosystem in PCNSL. T-cell exhaustion is a central feature of the tumor immune microenvironment and is characterized by sustained expression of inhibitory immune checkpoints, reduced effector function, and impaired cytotoxic activity [11]. In many tumors, these dysfunctional T-cell states are closely associated with an immunosuppressive niche shaped by both tumor cells and innate immune components [29]. In PCNSL, the CNS-restricted immune context further complicates T-cell regulation, where spatial and functional tumor heterogeneity may contribute to variable immune infiltration and T-cell dysfunction across regions [15, 30]. Although this study focuses on microglial reprogramming, it is plausible that microglia-derived immunosuppressive signals may indirectly contribute to T-cell exhaustion. Future studies incorporating comprehensive immune profiling will be required to define the interplay between microglia, tumor heterogeneity, and T-cell dysfunction in PCNSL.

However, a major limitation of our study is the lack of in-depth validation of the underlying molecular signaling pathways, as well as the absence of genetic knockout animal models to verify the functional role of ARG1 as a therapeutic target. Further mechanistic investigations, including pathway-specific analyses and ARG1-deficient models, will be required to clarify the molecular mechanisms underlying microglial remodeling and the antitumor effects of resveratrol.

Although our pharmacological inhibition experiments suggest that ARG1 may regulate microglial activation, at least in part through the PI3K–Akt signaling pathway, these findings should be interpreted with caution, as ARG1 was initially identified through transcriptomic and PPI network analyses. Future studies using microglia-specific genetic manipulation, such as conditional Arg1 knockout models, will be required to establish the causal role of ARG1 in microglial reprogramming and its contribution to PCNSL progression. Another limitation of the present study is that we did not evaluate the intrinsic immunological characteristics of PCNSL and DLBCL cells, such as the expression of MHC molecules, immune checkpoint proteins, or interferon responsiveness. Additionally, we did not directly assess the causal role of microglia in PCNSL progression using depletion strategies which warrants further investigation in future studies. Another limitation is that Iba1 and CD11b cannot completely distinguish resident microglia from infiltrating myeloid cells within the tumor microenvironment. Future studies employing more microglia-specific markers, such as TMEM119 or P2Y12, will enable more precise characterization of microglial populations. We acknowledge that pharmacological inhibition of Akt signaling was not performed in this study to further validate the PI3K–Akt axis, which warrants future investigation.

Overall, our findings not only provide preclinical evidence for resveratrol as a promising therapeutic candidate for PCNSL but also highlight the broader potential of modulating the CNS immune microenvironment as a promising strategy for treating this highly invasive lymphoma subtype.

## Supplementary Information

Supplementary Material 1: Figure S1 Immunofluorescence staining of KI67 in distance regions. (a), Representative immunofluorescence images from brain sections showing Ki-67 and Iba-1 in distance regions. Scale bar, 20 µm. (b), Representative immunofluorescence images from brain sections showing Ki-67 and Iba-1 in the tumor core regions. Scale bar, 20 µm. (c), Quantification of Ki-67 intensity and area in the indicated regions. (d) (e), Quantification of Ki-67 intensity and area in tumor core regions. Data are presented as mean ± SEM (n = 5 sections per group; 3 biological replicates). Statistical significance was determined using one-way ANOVA followed by Tukey’s multiple comparisons test. *P < 0.05, **P < 0.01, ***P < 0.001, ****P < 0.0001. (f) Representative immunofluorescence images from brain sections in negative control group showing tumor-free regions. (g) Representative immunofluorescence images from brain sections of intracranial model showing PCNSL tumor core regions. The tumor region is outlined by white dashed lines. Scale bar, 100 µm. (h) Representative immunofluorescence images from brain sections of intracranial PCNSL model showing tumor invasiveness. Scale bar, 50 µm. (i), Reconstruction error analysis for selection of the optimal NMF rank. (j), Hierarchical clustering and correlation analysis of NMF modules. (k), Marginal reduction in residual sum of squares (RSS) across candidate NMF ranks.

Supplementary Material 2: Figure S2 3D reconstruction and skeletonization of divergent microglia dynamic. (a) (b) (c) Representative image of 3D reconstruction and skeletonization in different regions in U2932 group. (d) (e) (f) Representative image of 3D reconstruction and skeletonization in different regions in HKBML group. Left: ImageJ-based skeletonization. Middle: 3D reconstruction shown in oblique (top) and lateral (bottom) views. Right: Frontal view of the reconstructed microglia. Scale bar, 5 µm. (g) Representative illustration of region selection for quantitative analysis. Schematic example showing the definition of the tumor core and distant regions used in this study. The core region was defined as the CD20-positive tumor mass, while the distant region was defined as a CD20-negative area located more than 2000 µm away from the tumor boundary, typically in the contralateral or tumor-free brain region. The tumor region is outlined by white dashed lines. (h) Quantification of branch number, average branch length, and end-point voxels of microglia in the core region of indicated group. Data are presented as mean ± SEM (n = 30 cells; 3 mice biological replicates, with 10 microglia randomly selected per section). Statistical significance was determined using one-way ANOVA followed by Tukey’s multiple comparisons test. *P < 0.05, **P < 0.01, ***P < 0.001, ****P < 0.0001.

Supplementary Material 3: Figure S3 Resveratrol and related compounds. (a), Chemical structures of resveratrol and related small-molecule compounds extracted from Ganoderma lucidum, as identified by mass spectrometry analysis. (b), CCK-8 assay showing the effects of these compounds on cell viability after 48 h treatment. Data are presented as mean ± SEM (n = 3).

Supplementary Material 4: Figure S4 Resveratrol colocalizes with microglia in vivo and exhibits binding affinity to ARG1. (A) Representative immunofluorescence images showing the colocalization of resveratrol with microglia in vivo. Microglia are labeled with (Iba1, red), and resveratrol signal is detected by (GFP, green). Merged images indicate spatial overlap. (B) Two-dimensional schematic representation of the molecular docking between resveratrol and ARG1, illustrating key interacting residues and binding interactions. (C–E) Three-dimensional molecular docking models depicting the interaction between resveratrol and ARG1 from different orientations, highlighting the binding pocket and spatial conformation.

Supplementary Material 5: Figure S5 Pro-inflammatory microglial activation in the tumor microenvironment of HKBML and DLBCL. (a)(b)(c)(d), Representative immunofluorescence images from core region or distance region in HKBML/DLBCL group showing microglia in the tumor core region. Scale bar, 20 µm.(e)(f), Quantitative analysis of iNOS-positive area and integrated fluorescence intensity. Data are presented as mean ± SEM (n = 5 sections per group; 3 biological replicates). Statistical significance was determined using one-way ANOVA followed by Tukey’s multiple comparisons test. *P < 0.05, **P < 0.01, ***P < 0.001, ****P < 0.0001.

Supplementary Material 6: Figure S6 Immunosuppressive microglial activation in the tumor microenvironment of HKBML and DLBCL. (a)(b)(c)(d), Representative immunofluorescence images from core region or distance region in HKBML/DLBCL group showing microglia in the tumor core region. Scale bar, 20 μm.(e)(f ), Quantitative analysis of CD206-positive area and integrated fluorescence intensity. Data are presented as mean ± SEM (n = 5 sections per group; 3 biological replicates). Statistical significance was determined using one-way ANOVA followed by Tukey’s multiple comparisons test. *P < 0.05, **P < 0.01, ***P < 0.001, ****P < 0.0001.

Supplementary Material 7: Figure S7 Microglial activation and polarization remodel the tumor microenvironment via PI3K–AKT-dependent cytokine secretion in vivo. (a), Representative immunofluorescence images showing microglial activation and polarization markers under different conditions. Cells were stained for microglial markers and polarization-associated proteins (iNOS, M1 subtype). Scale bar, 25 μm. (b), Representative immunofluorescence images showing microglial activation and polarization markers under different conditions. Cells were stained for microglial markers and polarization-associated proteins (CD206, M2 subtype). Scale bar, 25 μm. (c), Representative immunofluorescence images showing ARG1 expression in microglia under different conditions. Scale bar, 25 μm. (d), Quantitative analysis of fluorescence intensity in above immunofluorescence images under different conditions. Data are presented as mean ± SEM (n = 3 sections per group; 3 biological replicates). (e), Heatmap showing differential gene expression profiles in HKBML group microglia. (f ), Gene set enrichment upregulated biological processes in HKBML group microglia. (g), KEGG pathway enrichment analysis identifying PI3K–AKT signaling as a key regulatory pathway HKBML group microglia.(h), qPCR validation of PI3K–AKT signaling upgrade. Statistical significance was determined using one-way ANOVA followed by Tukey’s multiple comparisons test. *P < 0.05, **P < 0.01, ***P < 0.001, ****P < 0.0001. (i), Quantitative analysis of cytokine levels in co-culture cell model measured by ELISA. Data are presented as mean ± SEM (n = 6 biological replicates per group). Statistical significance was determined using one-way ANOVA followed by Tukey’s multiple comparisons test. *P < 0.05, **P < 0.01, ***P < 0.001, ****P < 0.0001.

Supplementary Material 8: Figure S8. Quantification and immunofluorescence colocalization analysis of ARG1⁺ and IBA1⁺ cells. (a), Representative immunofluorescence images showing the colocalization of ARG1 and IBA1 in different groups. From left to right: merged image, IBA1 (green), ARG1 (purple), DAPI (blue), and the processed image showing colocalized cells. (b), Quantification of the proportion of colocalized cells (ARG1+&IBA1+) among microglia and the number of IBA1⁺ microglia in different regions. Data are presented as mean ± SEM (n = 6 sections per group; 3 biological replicates).Statistical significance was determined using one-way ANOVA followed by Tukey’s multiple comparisons test. *P < 0.05, **P < 0.01, ***P < 0.001, ****P < 0.0001.

## Data Availability

No datasets were generated or analysed during the current study.
